# Polygenic risk score of metabolic dysfunction-associated steatotic liver disease amplifies the health impact on severe liver disease and metabolism-related outcomes

**DOI:** 10.1186/s12967-024-05478-z

**Published:** 2024-07-12

**Authors:** Lushan Xiao, Yan Li, Chang Hong, Pengcheng Ma, Hongbo Zhu, Hao Cui, Xuejing Zou, Jiaren Wang, Ruining Li, Jingzhe He, Shengxing Liang, Zeyang Li, Lin Zeng, Li Liu

**Affiliations:** 1 Department of Health Management, Nanfang Hospital, Southern Medical University, Guangzhou, 510515 China; 2 Guangdong Provincial Key Laboratory of Viral Hepatitis Research, Department of Infectious Diseases, Nanfang Hospital, Southern Medical University, Guangzhou, 510515 China; 3 Big Data Center, Nanfang Hospital, Southern Medical University, Guangzhou, 510515 China; 4 Department of Medical Oncology, The First Affiliated Hospital, Hengyang Medical School, University of South China, Hengyang, 421001 Hunan China; 5 School of Public Health, Southern Medical University, Guangzhou, 510515 China; 6 School of Health Management, Southern Medical University, Guangzhou, 510515 China

**Keywords:** Metabolic dysfunction-associated steatotic liver disease, Non-alcoholic fatty liver disease, Genome-wide association study, Biological variation

## Abstract

**Background:**

Although the inherited risk factors associated with fatty liver disease are well understood, little is known about the genetic background of metabolic dysfunction-associated steatotic liver disease (MASLD) and its related health impacts. Compared to non-alcoholic fatty liver disease (NAFLD), MASLD presents significantly distinct diagnostic criteria, and epidemiological and clinical features, but the related genetic variants are yet to be investigated. Therefore, we conducted this study to assess the genetic background of MASLD and interactions between MASLD-related genetic variants and metabolism-related outcomes.

**Methods:**

Participants from the UK Biobank were grouped into discovery and replication cohorts for an MASLD genome-wide association study (GWAS), and base and target cohorts for polygenic risk score (PRS) analysis. Autosomal genetic variants associated with NAFLD were compared with the MASLD GWAS results. Kaplan–Meier and Cox regression analyses were used to assess associations between MASLD and metabolism-related outcomes.

**Results:**

Sixteen single-nucleotide polymorphisms (SNPs) were identified at genome-wide significance levels for MASLD and duplicated in the replication cohort. Differences were found after comparing these SNPs with the results of NAFLD-related genetic variants. MASLD cases with high PRS had a multivariate-adjusted hazard ratio of 3.15 (95% confidence interval, 2.54–3.90) for severe liver disease (SLD), and 2.81 (2.60–3.03) for type 2 diabetes mellitus. The high PRS amplified the impact of MASLD on SLD and extrahepatic outcomes.

**Conclusions:**

High PRS of MASLD GWAS amplified the impact of MASLD on SLD and metabolism-related outcomes, thereby refining the process of identification of individuals at high risk of MASLD. Supplementation of this process with relevant genetic backgrounds may lead to more effective MASLD prevention and management.

**Supplementary Information:**

The online version contains supplementary material available at 10.1186/s12967-024-05478-z.

## Background

Non-alcoholic fatty liver disease (NAFLD) is the leading cause of cirrhosis and hepatocellular carcinoma (HCC), occurring in 25% of the global population [1]. Over the past ten years, significant conceptual advances have been made in understanding the complex pathophysiological mechanisms of this highly prevalent liver condition [2]. It has been progressively recognized that NAFLD is a multisystem disease where insulin resistance and related metabolic dysfunction play a critical pathogenic role [3]. NAFLD is associated with several liver-related morbidities, including cirrhosis, liver failure and HCC and extrahepatic complications, such as cardiovascular disease (CVD), type 2 diabetes mellitus (T2DM), chronic kidney disease (CKD) [4–6]. Recently, after a Delphi consensus process engaged global experts, the new term “metabolic dysfunction-associated steatotic liver disease” (MASLD) was proposed to redefine NAFLD [7]. As interpreted in the Delphi consensus, the clinical characteristics and diagnostic methods of MASLD performed differently from those of NAFLD, with no stigmatization. Compared to NAFLD, which is diagnosed by exclusion criteria, MASLD defined by the presence of hepatic steatosis in the context of co-existing cardiometabolic risk factors like elevated body mass index (BMI), insulin resistance, hypertension or dyslipidaemia [8].

In addition to clinical factors [9–12], genetic background profoundly influences fatty liver disease (FLD) and its related outcomes, as previous studies have revealed the associations between inherited risk factors and FLD [13, 14]. One such study demonstrated that FLD-related genetic variants amplified the health impact of metabolic dysfunction-associated fatty liver disease (MAFLD) [4]. However, this study applied the genetic variants from previous genome-wide association studies (GWAS) of FLD directly. Additionally, over the past few years, GWAS have revealed more than five single-nucleotide polymorphisms (SNPs) linked to the occurrence and development of NAFLD (e.g., PNPLA3, TM6SF2, MBOAT7, and GCKR); these are classic SNPs that have been well-explored in several independent studies [15, 16].

However, phenotypic variations may lead to differences in genetics, and differences in diagnostic criteria and clinical features exist between NAFLD and MASLD [7]. Although previous studies have identified genetic variants related to NAFLD [17], hepatic lipid accumulation [18, 19], liver enzymes, and various forms of liver diseases [20, 21], little is known regarding the genetic background of MASLD and its health impacts. Therefore, to further explore the progression of MASLD and its impact on severe health outcomes such as severe liver diseases (SLD), coronary artery disease (CAD), and other extrahepatic outcomes, it is necessary to understand the related genetic determinants.

Thus, in this study, we aimed to report a large MASLD GWAS by analyzing the epidemiological and genetic data of the UK Biobank (UKBB) to further assess the genetic background of MASLD and the interactions between MASLD-related gene variants and metabolism-related outcomes.

## Methods

### Study population

This investigation was conducted using the UKBB resource (application number: 92668). The UKBB is a prospective population-based cohort study that contains data from over 0.5 million participants aged 40–69 years. This data was collected between 2006 and 2010 and contains combined extensive measurements of baseline and genotype data. To assess the genetic background of MASLD, and the interactions between MASLD-related genetic variants and metabolism-related outcomes, we conducted a GWAS for 165,984 MASLD cases and 269,322 controls. Participants were randomly grouped into two cohorts for the GWAS: the discovery (n = 304,714) and replication cohorts (n = 130,592). For polygenic risk score (PRS) analysis, the discovery cohort was assigned as the base cohort, and the replication cohort served as the targeted cohort (Table 1).

**Table 1:** Study participants’ characteristics in the GWAS

Characteristic	Total (n = 435,306)	Discovery cohort (n = 304,714)	Replication cohort (n = 130,592)	*P*
Age, years	58 (50, 63)	58 (50, 63)	58 (50, 63)	0.254
Sex, n (%)				0.307
Female	236,195 (54.26)	165,182 (54.21)	71,013 (54.38)	
Male	199,111 (45.74)	139,532 (45.79)	59,579 (45.62)	
MASLD, n (%)	166,550 (38.26)	116,509 (38.24)	50,041 (38.32)	0.608
BMI, kg/m^2^	26.71 (24.13, 29.86)	26.72 (24.12, 29.85)	26.71 (24.13, 29.86)	0.703
WC, cm	90 (80, 99)	90 (80, 99)	90 (80, 99)	0.990
Glucose, mmol/L	4.93 (4.60, 5.31)	4.93 (4.60, 5.31)	4.93 (4.60, 5.31)	0.080
HBA1c, mmol/mol	35.1 (32.7, 37.7)	35.1 (32.7, 37.7)	35.1 (32.7, 37.7)	0.556
TG, mmol/L	1.49 (1.05, 2.15)	1.49 (1.05, 2.15)	1.49 (1.05, 2.16)	0.101
CHO, mmol/L	5.67 (4.93, 6.44)	5.67 (4.93, 6.44)	5.67 (4.92, 6.44)	0.765
LDL, mmol/L	3.53 (2.96, 4.13)	3.53 (2.96, 4.13)	3.53 (2.96, 4.13)	0.503
HDL, mmol/L	1.4 (1.18, 1.68)	1.4 (1.18, 1.68)	1.4 (1.17, 1.68)	0.734
GGT, U/L	26.2 (18.4, 40.8)	26.1 (18.4, 40.8)	26.2 (18.5, 40.9)	0.231
CRP, mg/L	1.32 (0.66, 2.74)	1.32 (0.65, 2.74)	1.33 (0.66, 2.76)	0.208
Medication usage				
Antihypertensive drugs, n (%)	97,873 (22.48)	68,266 (22.4)	29,607 (22.67)	0.053
Hypoglycemic drugs, n (%)	14,737 (3.39)	10,286 (3.38)	4451 (3.41)	0.591
Statins, n (%)	77,376 (17.78)	54,187 (17.78)	23,189 (17.76)	0.840

*GWAS* genome wide association study, *MASLD* metabolic dysfunction-associated steatotic liver disease, *BMI* body mass index, *WC* waist circumference, *HbA1c* glycated hemoglobin, *TG* plasma triglycerides, *CHO* plasma total cholesterol, *LDL* plasma low-density lipoprotein cholesterol, *HDL* plasma high-density lipoprotein cholesterol, *GGT* gamma-glutamyl transferase, *CRP* C-reactive protein

### Diagnosis of MASLD

For the UKBB MASLD cohort, we calculated the fatty liver index (FLI) for each participant and defined hepatic steatosis as FLI ≥ 60 [22], as imaging or histological data of liver were not available. The diagnostic criteria of MASLD were set with reference to the Delphi consensus. Briefly, MASLD was diagnosed based on FLI-diagnosed hepatic steatosis and presence of one of the following four criteria: (1) overweight or obesity, (2) diagnosis of T2DM or prediabetes, (3) hypertension, or (4) dyslipidemia [7, 23].

### Diagnosis of NAFLD

NAFLD was defined based on the International Classification of Diseases (ICD) Ninth and Tenth Revision codes from in-patient hospital diagnoses (Table S1). The diagnostic criteria required evidence of hepatic steatosis in the absence of significant alcohol consumption (< 30 g per day for men and < 20 g per day for women). Additional exclusion criteria included other causes of liver fat accumulation such as viral hepatitis, medication use, or other chronic liver diseases.

### GWAS and PRS analyses

In the discovery cohort, a total of 304,714 Caucasian British individuals (165,182 females and 139,532 males) with genotype data meeting MASLD diagnosis criteria were analyzed for GWAS, comprising 116,509 MASLD cases. In the replication cohort, a total of 130,592 Caucasian British individuals comprising 50,041 MASLD cases were analyzed for GWAS (Fig. S1). For NAFLD, 301,846 Caucasian British individuals comprising 3881 NAFLD cases were analyzed.

Two very similar genotyping arrays (Affymetrix UK BiLEVE and UK Biobank Axiom arrays) were used for genotyping participants in the UK Biobank, and imputation was performed using the merged UK10K and 1000 Genomes phase 3 reference panels [24]. Variants was restricted to high-quality autosomal variants with a minor allele frequency > 0.1%, minor allele count > 5%, info score > 0.3, genotype hard call rate > 0.95, and Hardy–Weinberg *P* > 1 × 10^–6^. Finally, a total of 12,250,143 and 12,248,938 SNPs were included in the GWAS in the discovery and replication cohorts, respectively. We tested autosomal genetic variants for association with MASLD, assuming an additive allelic effect and using FastGWA-GLMM [25] implemented in Genome-wide Complex Trait Analysis software to account for population structure and cryptic relatedness. All models included the following covariates as fixed effects: sex, age, genotyping array, and principal components 1–20. The genome-wide significance threshold was set as 5 × 10^−8^. The genome-wide significant variants in the discovery cohort were extracted and analyzed in the replication cohort.

To detect multiple independent association signals at each genome-wide significant MASLD locus, we applied approximate conditional and joint genome-wide association analysis using the software package GCTA v1.91.14. Variants with high collinearity (multiple regression R^2^ > 0.9) were ignored, and those situated more than 1000 Kbp away were assumed to be independent. A reference sample of 50,000 unrelated White British individuals randomly selected from the UK Biobank was used to model linkage disequilibrium (LD) patterns between variants. The reference genotyping dataset comprised the same variants as those assessed in the GWAS. Conditionally independent variants reaching genome-wide significance were annotated to the physically closest gene using 3DSNPv1.0 [26].

We used LD score regression (LDSC) [27] to estimate the amount of genomic inflation present in the data due to residual population stratification, cryptic relatedness, and other latent sources of bias. PRSice-2 software (version 2.3.3 for R) was used to estimate the PRS using odds ratios (ORs) from GWAS data in the base cohort, and we estimated the individual PRS of MASLD phenotypes for the target cohort.

### Outcome data

ICD codes were used to define incident diseases (Table S1), and associations between MASLD and SLD, hypertension, CAD, stroke, heart failure (HF), CKD, T2DM, and overall survival were examined. SLD was defined as a composite diagnosis of cirrhosis, decompensated liver disease (i.e., esophageal varices with or without bleeding, portal hypertension, hepatorenal syndrome, and liver failure), HCC, and/or liver transplantation in any of the aforementioned records.

### Statistical analysis

Continuous data were summarized as means and standard deviations (SD) when normally distributed and as median and interquartile range when skewed; categorical data were summarized as frequencies and percentages. For comparison between groups, continuous data were assessed using the independent *t*-test or Mann–Whitney U test. In contrast, categorical data were evaluated using the chi-square or Fisher’s exact tests.

Cox proportional hazards models were used to assess the health impact of MASLD, and the hazard ratio (HR) derived from the three models was used to quantify the health impact. Model 1 was unadjusted. Model 2 was adjusted for sex, age at recruitment, genotyping chips, and BMI. Model 3 was further adjusted for hypoglycemic drugs, antihypertensive drugs and statins, based on model2 (Table S2). The hypoglycemic drugs included PPAR agonists, biguanides, alpha-glucosidase inhibitors, benzoic acid derivatives, sulfonylureas, and insulin [28]. The antihypertensive drugs include ACE inhibitors, calcium channel blockers, beta-blockers, thiazides, and angiotensin II receptor blockers [29].

We categorized the participants into low- and high-PRS groups using the midpoint of the PRS as the cut-off. The associations between MASLD and morbidities were reassessed by considering the PRS. All analyses were performed using R software (version 4.0.2; R Foundation for Statistical Computing, Vienna, Austria). For the GWAS, significance was set at *P* < 5 × 10^−8^; for other analyses, it was set at *P* < 0.05.

### Ethics statement

All participants provided informed consent via electronic signature at the baseline assessment. Ethical approval was granted for the use of the UK Biobank by the North West-Haydock Research Ethics Committee (REC reference: 16/NW/0274). The study protocol conformed to the ethical guidelines of the 1975 Declaration of Helsinki.

## Results

### Characteristics of study participants

A total of 435,306 participants of White British descent from the UK Biobank were selected for a GWAS of MASLD, which comprised 166,550 subjects diagnosed with MASLD (Table 1, Fig. S1). Table 1 presents the basic characteristics of the study participants. In the discovery cohorts, over half of the participants were women, and the median BMI indicated an overweight status (BMI ≥ 25 kg/m^2^). The demographic, clinical and biochemical parameters were similar between the discovery and replication cohorts (*P* > 0.05).

### MASLD case–control GWAS

**Fig. 1:**
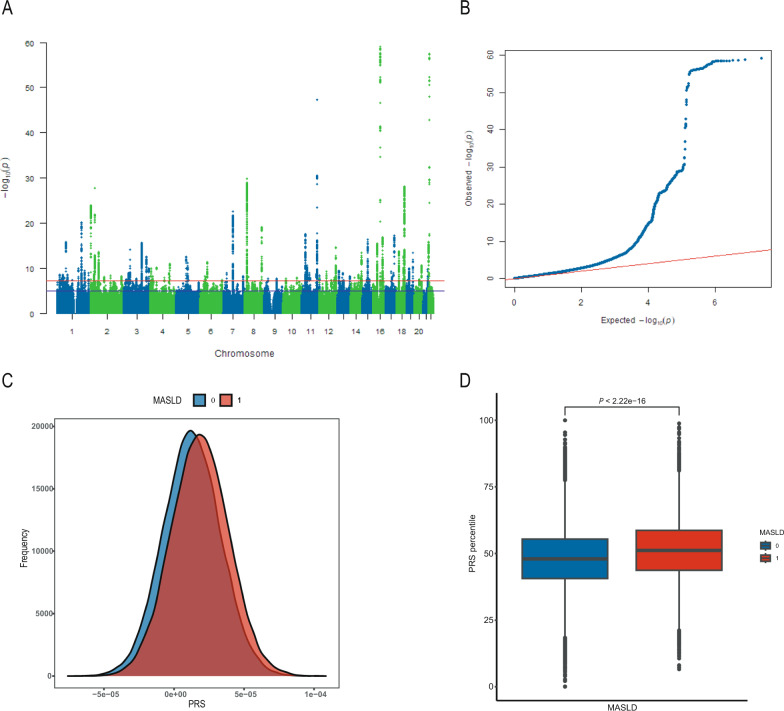
Results of GWAS case–control analysis and polygenic risk score (PRS). Manhattan plot (**A**) and Q–Q plot (**B**) of genome-wide markers for MASLD in the discovery cohort. **C** Density plots of the polygenic risk score between MASLD and non-MASLD groups. **D** Comparison of PRS percentile between two groups. NAFLD Biopsy: the summary statistics of GWAS for NAFLD diagnosed with biopsy. *MASLD* metabolic dysfunction-associated steatotic liver disease, *GWAS* genome-wide association study, *NAFLD* non-alcoholic fatty liver disease

To explore the genetic association of SNPs with phenotypes, we analyzed autosomal SNPs and identified 114 conditionally independent signals associated with MASLD mapping to loci at *P* < 5 × 10^–8^ (Table S3). The MASLD of the discovery cohort case–control analysis is presented as a Manhattan plot in Fig. 1A and a QQ plot of the association results is shown in Fig. 1B. Although there was substantial inflation of the test statistics (λ = 1.57), LD score regression indicated most of the inflation to be a result of polygenicity rather than population stratification (LD score regression intercept, 1.0598 (0.0142); ratio, 0.077). After OR analysis in the replication cohort for variants significantly related to MASLD found in the discovery, 16 conditionally independent SNPs were replicated as significantly related (*P* < 5 × 10^–8^; Table 2), located in the genes GCKR, LOC124905962, MON1A, MLXIPL, LPL, ZPR1, BDNF, FAIM2, EXOC3L4, FTO, APOBR, BP11-795H16.2, GGT1, and BCRP3.

**Table 2:** Summary of top conditionally independent SNPs in the MASLD case–control analysis

SNP	Chr	A1	Nearest gene	Discovery cohort		Replication cohort	
				OR (95% CI)	*P*	OR (95% CI)	*P*
2:27748992:AT:A	2	A	GCKR	0.938 (0.927–0.949)	1.855E−28	0.945 (0.929–0.961)	1.443E−10
rs6731688	2	C	–	1.079 (1.064–1.095)	8.908E−25	1.071 (1.047–1.095)	1.438E−09
rs62106258	2	C	LOC124905962	0.876 (0.854–0.898)	1.985E−24	0.856 (0.824–0.890)	4.093E−15
3:49959570:CA:C	3	C	MON1A	1.045 (1.034–1.057)	6.808E−15	1.049 (1.032–1.067)	2.696E−08
rs17145750	7	T	MLXIPL	0.927 (0.913–0.941)	2.859E−23	0.924 (0.903–0.945)	5.749E−12
rs2119690	8	A	LPL	0.932 (0.920–0.943)	1.332E−30	0.940 (0.923–0.957)	4.202E−11
rs11030108	11	G	BDNF/BDNF-AS	0.949 (0.938–0.960)	2.624E−18	0.948 (0.932–0.966)	6.171E−09
rs964184	11	C	ZPR1	0.887 (0.873–0.902)	4.245E−48	0.885 (0.863–0.907)	8.474E−23
rs7132908	12	A	FAIM2	1.036 (1.025–1.048)	5.292E−10	1.053 (1.035–1.071)	4.429E−09
rs2274685	14	G	EXOC3L4	1.044 (1.033–1.056)	3.529E−14	1.053 (1.035–1.071)	3.337E−09
rs11075985	16	A	FTO	1.097 (1.084–1.109)	8.278E−60	1.090 (1.072–1.109)	8.896E−24
rs40831	16	G	APOBR	1.040 (1.029–1.052)	4.192E−12	1.050 (1.033–1.068)	1.491E−08
18:57850927:GTCT:G	18	G	RP11-795H16.2	1.077 (1.063–1.091)	6.304E−29	1.085 (1.064–1.107)	3.038E−16
rs538303513	18	GAT	–	0.868 (0.835–0.902)	1.038E−12	0.831 (0.782–0.882)	1.143E−09
rs116946885	22	C	BCRP3	0.856 (0.826–0.886)	2.379E−18	0.840 (0.796–0.886)	1.769E−10
rs3859862	22	G	GGT1	1.099 (1.087–1.112)	3.364E−58	1.095 (1.076–1.114)	3.977E−24

*MASLD* metabolic dysfunction-associated steatotic liver disease, *SNP* single nucleotide polymorphism, *Chr* chromosome, *OR* odds ratio, *95% CI* 95% confidence interval

To further explore the 16 independent SNPs, we analyzed their association with survival, as well as their cell-specific differential effects. After adjusting for sex, age at recruitment, genotyping chips, and BMI, the independent SNPs were not associated with OS (Table S4–6). Additionally, MASLD GWAS was conducted within subgroups based on sex (male and female) and BMI categories (normal weight, overweight, and obese; Table S7–11, Fig. S2–3).

### Comparison of GWAS results between MASLD and NAFLD

We conducted a GWAS of NAFLD in the discovery cohort to compare the significant SNPs between MASLD and NAFLD, as well as a GWAS of NAFLD in the discovery cohort. In addition, we extracted previously reported SNPs associated with NAFLD diagnosed using biopsy [20] or imaging [30]. The details of the three NAFLD cohorts are shown in Table S12. The effects of 83 intersectional significant SNPs that reached genome-wide significance for the NAFLD imaging and NAFLD UKBB cohorts were identified; however, only 18 and 24 effect sizes estimated from the GWAS variants for these two cohorts were found in our discovery cohort of MASLD UKBB, respectively (Fig. S4A).

To further explore the distinction of significant genetic variants between MASLD and NAFLD, we annotated the significant SNPs among the GWAS results and compared them between the MASLD UKBB cohort and three NAFLD cohorts using various diagnostic methods. The results showed that the effect at PNPLA3, SAMM50, and PARVB loci reached genome-wide significance only in the NAFLD cohort but not in MASLD. However, other chromosomal loci including TM6SF2, SUGP1, PBX4, ZNF101, ZNF512, LOC124904656, HAPLN4, GATAD2A, C2orf16, and TRIB1 that were identified as associated with NAFLD in various NAFLD cohorts were significantly associated with MASLD (Fig. S4).

### Polygenic risk score for MASLD

We used a base cohort to estimate the OR related to MASLD and subsequently applied the OR to estimate PRS in the target cohort (n = 130,592) recruited from the replication cohort. The median PRS of all participants in the target cohort was 1.476e−05 (8.805e−07, 2.871e−05), and we identified 65,296 participants with a high PRS. As shown in Fig. 1C, the MASLD-PRS was normally distributed in both the MASLD and non-MASLD groups. The mean PRS was higher in the MASLD group than the non-MASLD group (*P* < 0.05), and the mean MASLD-PRS percentile (SD) in the non-MASLD group was 47.97 (11.06) vs 51.15 (11.11) in the MASLD group (*P* < 0.05; Fig. 1D). In addition, we generated receiver operating characteristic (ROC) curves of the PRS derived from different GWAS results in the target cohort, which provided a measure of their diagnostic power for MASLD. MASLD-PRS outperformed NAFLD-PRS in estimating MASLD status and show that MASLD-PRS achieved the highest area under the ROC curve (AUC = 0.581, 95% confidence interval (CI): 0.577–0.584). (Fig. S4C, D) External validation was performed using the Finngen dataset to calculate the NAFLD-PRS.

### PRS amplified the health impact of MASLD

**Fig. 2:**
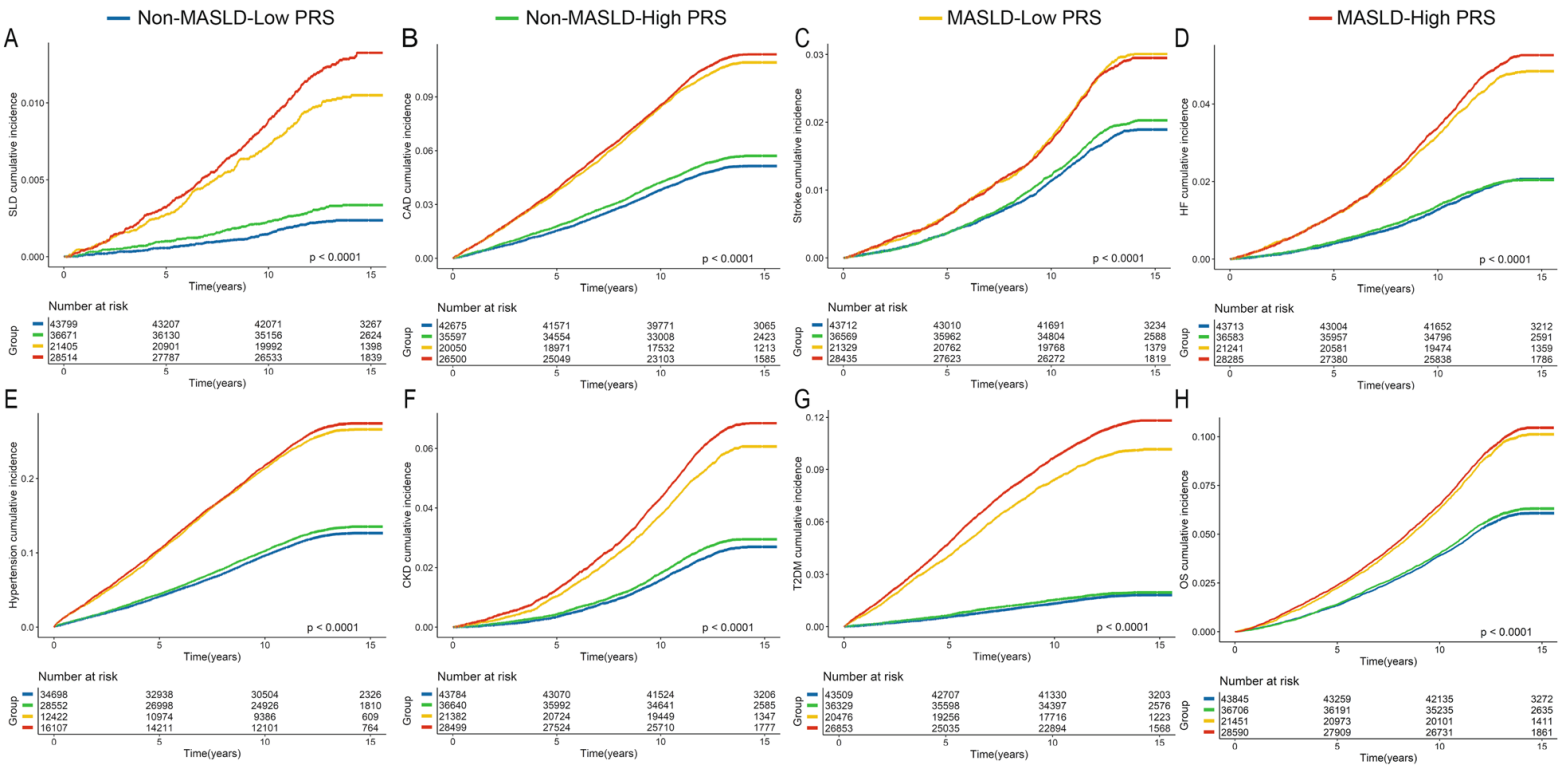
The cumulative risks of developing incident outcomes among the replication cohort, by MASLD and PRS. **A** SLD: severe liver disease, **B** CAD: coronary artery disease; **C** stroke; **D** HF: heart failure; **E** hypertension; **F** CKD: chronic kidney disease; **G** T2DM: type 2 diabetes mellitus; **H** death. Non-MASLD patients with low PRS were set as the reference group. *MASLD* metabolic dysfunction-associated steatotic liver disease, *PRS* polygenetic risk score

**Fig. 3:**
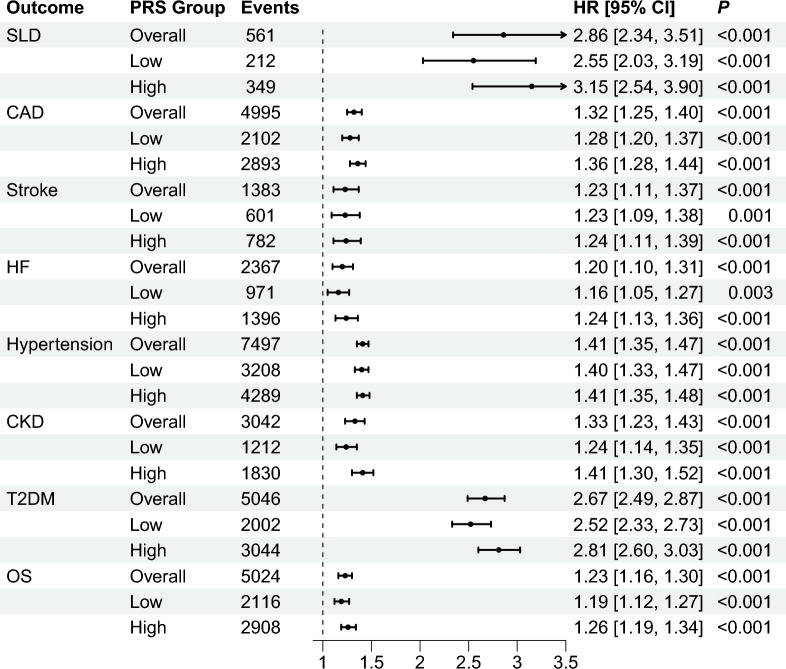
The impact of MASLD on SLD and extrahepatic outcomes. The hazard ratios were obtained from model 2 with the non-MASLD as the reference group. “Overall” refers to the whole MASLD participants without the consideration of PRS. “Low” and “High” denote the MASLD participants who had low and high PRS, respectively. *MASLD* metabolic dysfunction-associated steatotic liver disease, *PRS* polygenetic risk score, *SLD* severe liver disease, *CAD* coronary artery disease, *HF* heart failure, *CKD* chronic kidney disease, *T2DM* type 2 diabetes mellitus, *OS* overall survival

During a median follow-up of 13.8 years, we identified 779, 9083, 2869, 3939, 15,512, 5192, 6508, and 9893 incident events of SLD, CAD, stroke, HF, hypertension, CKD, T2DM, and death, respectively in the replication cohort. MASLD was associated with an increased risk of both SLD and extrahepatic comorbidities. To further verify the amplified effect of high PRS, we conducted Kaplan–Meier and Cox regression analyses. The impact of MASLD on SLD and extrahepatic morbidities was amplified by a high PRS, especially for SLD and T2DM (Figs. 2 and 3). In model 2, compared with those who were non-MASLD, MASLD participants and those with high PRS had a multivariate-adjusted HR for SLD of 3.15 (95% CI 2.54–3.90); for CAD, it was 1.36 (95% CI 1.28–1.44); for stroke, it was 1.24 (95% CI 1.11–1.39); for HF, it was 1.24 (95% CI 1.13–1.36); for hypertension, it was 1.41 (95% CI 1.35–1.48); for CKD, it was 1.41 (95% CI 1.30–1.52); for T2DM, it was 2.81 (95% CI 2.60–3.03); and for overall survival, it was 1.26 (95% CI 1.19–1.34; Table S2, Fig. 3). After further adjustment for medication usage, including hypoglycemic drugs, antihypertensive drugs, and statins for adjustment, these associations were unchanged (Model 3).

## Discussion

The GWAS in this study found 16 conditionally independent SNPs to be associated with MASLD at genome-wide significance levels. Differences were found when comparing the results of NAFLD-related genetic variants. Additionally, we constructed a MASLD PRS based on this GWAS and examined the association between the PRS and MASLD. We also revealed the impact of MASLD on SLD, with extrahepatic metabolism-related outcomes shown to be amplified by MASLD PRS.

The complexity of the MASLD phenotype is due to interactions between the components of metabolic syndrome and a genetic predisposition to environmental factors. Further research on the utility of MASLD as a sub-phenotype will strengthen its recognition within the field of fatty liver [31]. However, to our knowledge, few previous studies have explored the genetics of MASLD. He et al. explored the Mendelian randomization of MAFLD and iron status but did so by directly using the results of a previous GWAS of NAFLD [32]. This study’s results identified 16 variants primarily located in genes such as GCKR, MON1A, and LPL that were independently associated with MASLD at genome-wide significance levels and determined differences in genetic variants between MASLD and NAFLD.

Our study corroborated some previous identified variants associated with FLD, such as GCKR, LPL, FTO [17], and MLXIPL [33], which indicated the good validity of the data sources used in the current analysis. Additionally, it is essential to elucidate the variants found in our GWAS of MASLD. GWAS and multiple candidate gene studies have identified GCKR variants as being linked to several metabolic parameters, including triglyceride (TG) levels, insulin resistance, and fasting plasma glucose levels [34–36], as well as metabolic disorders like T2DM and dyslipidemia [37, 38]. While previous research has reported GCKR as a disease-predisposing variant for NAFLD [16], this study found an association between GCKR and a decreased risk of MASLD (OR = 0.938 [95% CI 0.927–0.949]. This association could be attributed to the stronger relationship between GCKR variants and improved glucose metabolism. A previous study found that the association between rs3812316 (MLXIPL) and alpha-linolenic acid intake as well as TG levels in Mexican Mestizo women [39], and Hehl et al. highlighted the link between MLXIPL variants and lower serum TG and apolipoprotein-B levels [33]. LPL variants have also been shown to significantly contribute to dyslipidemia, being associated with several conditions including obesity, metabolic syndrome, and atherosclerosis [40–43]. Additionally, the rs964184 (ZPR1) has been reported to be associated with variations in lipid levels [44], as well as metabolic disorders such as NAFLD, T2DM, and CVD [45–47]. Notably, after stratified analysis of the MASLD GWAS by BMI categories or sex, rs964184 (ZPR1) remained significant across all subgroups, highlighting its potential as a biomarker and therapeutic target for effective MASLD management. The rs7132908 (FAIM2) was found to be significantly associated with obesity [48, 49], and FTO was the first GWAS-identified obesity gene [50]. Therefore, these variants might be potential pharmacological targets for treatment of hyperlipidemia and MASLD, especially for high-risk patients.

Moreover, although there have been fewer studies exploring the relationship between BDNF and metabolic disorders, there is evidence of an association between BDNF and the reduction in BMI, waist circumference, glucose, insulin, and risk for T2DM, mainly in Asian populations [51, 52]. MON1A has been identified as having a critical role in controlling macrophage iron metabolism [53], and a growing body of evidence has suggested that macrophage infiltration in adipose tissue causes inflammation and cytokine production and contributes to the development of metabolic decompensation, insulin resistance, and T2DM [54, 55]. GWAS revealed an association between GGT1 and plasma levels of liver enzymes, as well as alcohol-associated liver disease [56, 57]. The role of LOC124905962, EXOC3L4, BP11-795H16.2, and BCRP3 in metabolic disorders has not been well-documented yet. To further investigate the underlying mechanisms, we explored the cell-type-specific expression patterns of these genes using single-cell RNA sequencing data from liver cells in metabolic syndrome mouse models (Fig. S5) [58]. This analysis was conducted using online tools available at the Single Cell Portal (SCP1404). The findings revealed that GCKR and MLXIPL are highly expressed in pericentral and periportal hepatocytes, LPL is specific to Kupffer cells, and MON1A is predominantly expressed in T/NK cells. Currently, there is no MASLD-specific single-cell RNA sequencing dataset available, which underscores a potential area for future research. Overall, more mechanistic studies are necessary to gain a deeper understanding of the role of these variants in metabolic pathways and in the development of steatosis.

Sex and BMI were found to be strongly correlated with the incidence of MASLD [10, 59, 60]. Therefore, we conducted MASLD GWAS within subgroups stratified by sex and BMI categories. In the sex subgroup analysis, SNPs such as rs10889356 (DOCK7) and rs72836561 (CD300LG) showed significant associations exclusively in males, highlighting the roles of lipid and glucose metabolism [61, 62], while SNPs such as rs545608 (SEC16B), previously proven to be female-specific, was exclusively found in the female group [63]. The presence of these SNPs suggests potential gender-specific genetic influences on MASLD. For the BMI subgroups, the identification of significant SNPs such as rs17145750 (MLXIPL) in the normal BMI population suggests that lipid metabolism is also pivotal in the development of MASLD among lean individuals [39]. Further research is needed to validate the identified specific-SNPs and explore their functional implications. These findings underscore the need to consider demographic differences, particularly sex and BMI, in the development of personalized diagnostic and therapeutic strategies.

We constructed the genome-wide PRS and further assessed the interactions between the PRS and health outcomes. Previous studies have reported genetic variations associated with an increased risk of liver disease progression and adverse extrahepatic outcomes [64–66]. For example, Liu et al. demonstrated that the genetic risk score derived from FLD-associated variants increases the risk of hepatic events and extrahepatic outcomes [4]. In our study, the GWAS-based PRS of MASLD was shown to have better performance in identifying MASLD than that based on a GWAS of NAFLD, indicating the difference in genetic backgrounds between MASLD and NAFLD. Further analysis showed that PRS derived from GWAS of MASLD amplified the effect of MASLD on SLD and metabolism-related outcomes such as T2DM, CAD, stroke, HF, and CKD, thus complementing the findings from a genetic perspective. Both intrahepatic and extrahepatic outcomes indicate that individuals with MASLD in the high-PRS group are at an increased risk of disease progression. This finding highlights the potential of the PRS to predict MASLD progression. These findings are essential for preventing and managing metabolism-related diseases in patients with MASLD. Future research should focus on longitudinal studies to validate these findings and establish the PRS as a reliable marker for disease monitoring and management. The inclusion of additional cohorts and extended follow-up periods will be essential to the constancy of the PRS across diverse populations.

Our study has several limitations. First, it only included individuals from Caucasian British ethnic backgrounds, compromising the generalizability of its results to other ethnicities. In addition, MASLD is a common phenotype in the European population; thus, our population controls cannot be considered entirely free of MASLD, and there is no known way of investigating this further. Finally, we used serum biomarkers to diagnose fatty liver, but not liver biopsy or imaging data. Although the diagnosis of steatotic liver disease requires supporting biopsy imaging by definition of MASLD, FLI remains a useful diagnostic biomarker for FLD with acceptable accuracy and is widely used in population-based studies.

## Conclusions

This study is the first to combine GWAS and PRS to identify the genetic components of MASLD. We found that high PRS amplified the health of patients with MASLD, especially those with SLD and T2DM. Therefore, the construction of PRS may help identify individuals at high risk of MASLD and metabolism-related outcomes. Supplementation of this process with MASLD-related genetics information may lead to a more accurate prediction of disease progression and more effective management of MASLD.

### Supplementary Information

Supplementary Material 1: Fig. S1. Study flow chart. MASLD: metabolic dysfunction-associated steatotic liver disease; GWAS: genome-wide association study.

Supplementary Material 2: Fig. S2. Results of GWAS case–control analysis within subgroups based on sex. Manhattan plot (A) and Q–Q plot (B) of genome-wide markers for MASLD in the discovery cohort among male participants. Manhattan plot (C) and Q–Q plot (D) of genome-wide markers for MASLD in the discovery cohort among female participants. MASLD: metabolic dysfunction-associated steatotic liver disease; GWAS: genome-wide association study.

Supplementary Material 3: Fig. S3. Results of GWAS case–control analysis within subgroups based on BMI categories. Manhattan plot (A) and Q–Q plot (B) of genome-wide markers for MASLD in the discovery cohort among the participants with normal BMI. Manhattan plot (C) and Q–Q plot (D) of genome-wide markers for MASLD in the discovery cohort among overweight participants. Manhattan plot (E) and Q–Q plot (F) of genome-wide markers for MASLD in the discovery cohort among obese participants. MASLD: metabolic dysfunction-associated steatotic liver disease; GWAS: genome-wide association study; Normal BMI: BMI < 25 kg/m^2^; overweight: 25 kg/m^2^ ≤ BMI < 30 kg/m^2^; obese: BMI ≥ 30 kg/m^2^.

Supplementary Material 4: Fig. S4. Comparison of GWAS and PRS results between MASLD and NAFLD. (A) The intersection of significant SNPs associated with MASLD and published SNPs associated with NAFLD. (B) Comparison of MASLD and NAFLD-related genes. (C) and (D) ROC graphical plot the diagnostic ability of different sources of PRS for MASLD in the replication cohort. NAFLD Biopsy: the summary statistics of GWAS for NAFLD diagnosed with biopsy; NAFLD Image: the summary statistics of GWAS for NAFLD diagnosed with image; NAFLD UKBB: Summary statistics of GWAS for NAFLD in the UKBB discovery cohort; MASLD UKBB: Summary statistics of the GWAS for MASLD in the discovery cohort of UKBB; MASLD: metabolic dysfunction-associated steatotic liver disease; GWAS: genome-wide association study; NAFLD: non-alcoholic fatty liver disease; UKBB: UK Biobank.

Supplementary Material 5: Fig. S5. Cell type-specific expression of the genes identified in MASLD GWAS based on the single-cell RNA sequencing data from liver cells in metabolic syndrome mouse models. (A) Dot plots illustrating the expression of genes in each cell cluster. (B) UMAP plot of 11 cell clusters. (C–L) UMAP plots showing the expression of specific genes (GCKR, MON1A, MLXIPL, LPL, BDNF, FAIM2, EXOC3L4, FTO, APOBR, and GGT1) across the cell clusters. UMAP: Uniform Manifold Approximation and Projection. This analysis was conducted using online tools available at the Single Cell Portal. (https://singlecell.broadinstitute.org/single_cell/study/SCP1404/multitissue-single-cell-analysis-reveals-differential-tissue-cellular-and-molecular-sensitivity-between-fructose-and-high-fat-high-sucrose-diets-liver).

Supplementary Material 6: Table S1. Coding algorithms for defining diseases in the UK Biobank.

Supplementary Material 7: Table S2. The associations of MASLD in different PRS group with SLD and extrahepatic outcomes.

Supplementary Material 8: Table S3. Summary of all conditionally independent SNPs in the MASLD case–control analysis.

Supplementary Material 9: Table S4. The associations of top conditionally independent SNPs with overall survival in the whole cohort.

Supplementary Material 10: Table S5. The associations of top conditionally independent SNPs with overall survival in the MASLD group.

Supplementary Material 11: Table S6. The associations of top conditionally independent SNPs with overall survival in the non-MASLD group.

Supplementary Material 12: Table S7. Summary of conditionally independent SNPs in the MASLD case–control analysis among male participants.

Supplementary Material 13: Table S8. Summary of conditionally independent SNPs in the MASLD case–control analysis among female participants.

Supplementary Material 14: Table S9. Summary of conditionally independent SNPs in the MASLD case–control analysis among participants with normal BMI.

Supplementary Material 15: Table S10. Summary of conditionally independent SNPs in the MASLD case–control analysis among overweight participants.

Supplementary Material 16: Table S11. Summary of conditionally independent SNPs in the MASLD case–control analysis among obese participants.

Supplementary Material 17: Table S12. Characteristics of NAFLD GWAS included in our study.

## Data Availability

The UK Biobank data used in this study is publicly available online (www.ukbiobank.co.uk; application number 92668). The datasets used and/or analyzed during the current study are available from the corresponding author on reasonable request.

## Abbreviations

BMI: Body mass index
CAD: Coronary artery disease
CVD: Cardiovascular disease
CKD: Chronic kidney disease
ESRD: End-stage renal disease
FLD: Fatty liver disease
GWAS: Genome-wide association study
HCC: Hepatocellular carcinoma
HF: Heart failure
HR: Hazard ratio
ICD: International Classification of Diseases
LD: Linkage disequilibrium
LDSC: LD score regression
MAFLD: Metabolic dysfunction-associated fatty liver disease
MASLD: Metabolic dysfunction-associated steatotic liver disease
NAFLD: Non-alcoholic fatty liver disease
OR: Odds ratio
PRS: Polygenic risk score
SLD: Severe liver disease
SNPs: Single-nucleotide polymorphisms
T2DM: Type 2 diabetes mellitus
UKBB: UK Biobank
WC: Waist circumference
