# Supplementary material for: Polygenic risk score of metabolic dysfunction-associated steatotic liver disease amplifies the health impact on severe liver disease and metabolism-related outcomes
Source: J Transl Med. 2024 Jul 12;22:650. doi: 10.1186/s12967-024-05478-z (PMC11241780; doi:10.1186/s12967-024-05478-z)
Supplement: Supplementary file 1 — Supplementary Material 1: Fig. S1. Study flow chart. MASLD: metabolic dysfunction-associated steatotic liver disease; GWAS: genome-wide association study. [file 12967_2024_5478_MOESM1_ESM.pdf]

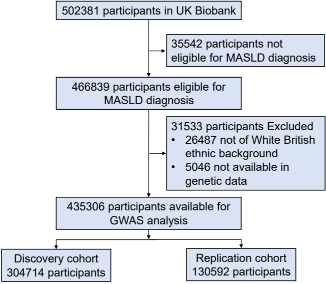
502381 participants in UK Biobank
35542 participants not eligible for MASLD diagnosis
466839 participants eligible for MASLD diagnosis
31533 participants Excluded
• 26487 not of White British ethnic background
• 5046 not available in genetic data
435306 participants available for GWAS analysis
Discovery cohort 304714 participants
Replication cohort 130592 participants
