# Supplementary figures and images for: Polygenic risk score of metabolic dysfunction-associated steatotic liver disease amplifies the health impact on severe liver disease and metabolism-related outcomes
Source: J Transl Med. 2024 Jul 12;22:650. doi: 10.1186/s12967-024-05478-z (PMC11241780; doi:10.1186/s12967-024-05478-z)

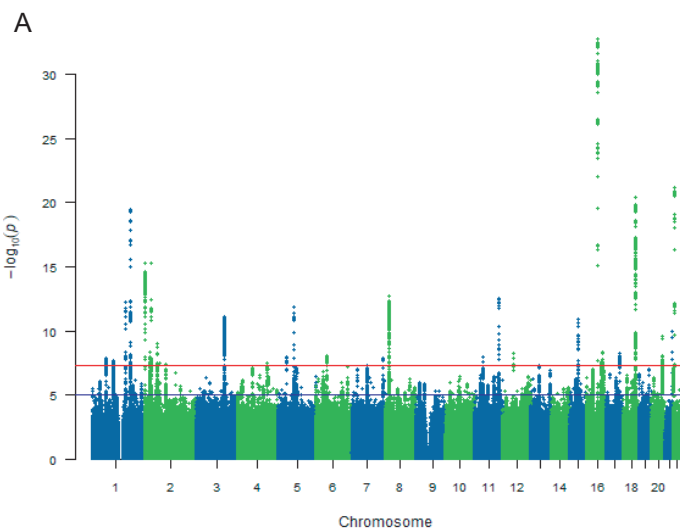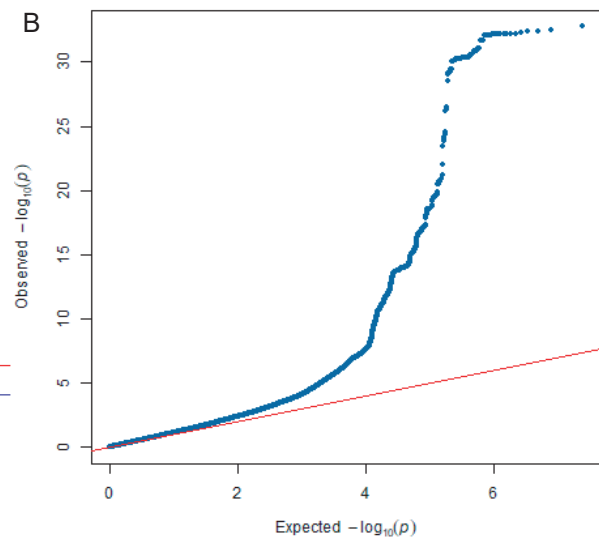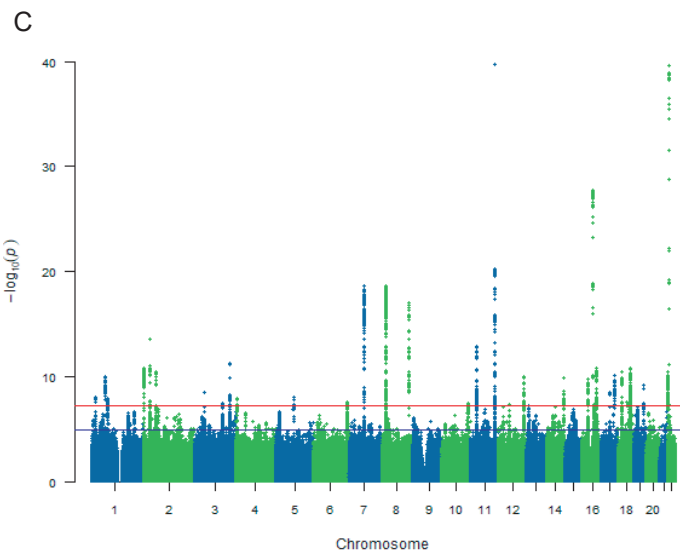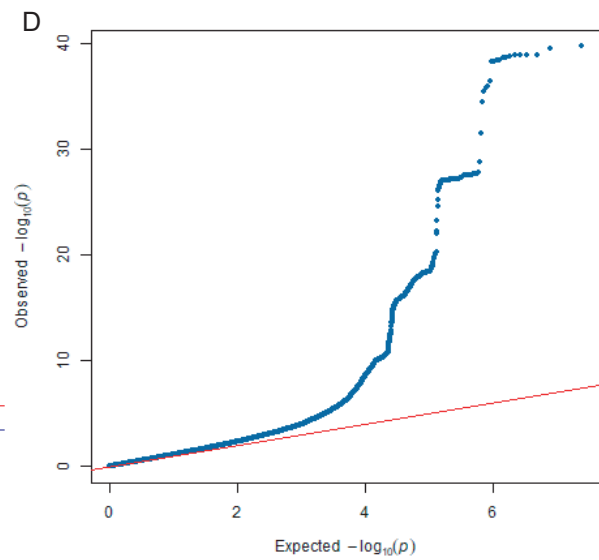

Supplement: Supplementary file 2 — Supplementary Material 2: Fig. S2. Results of GWAS case–control analysis within subgroups based on sex. Manhattan plot (A) and Q–Q plot (B) of genome-wide markers for MASLD in the discovery cohort among male participants. Manhattan plot (C) and Q–Q plot (D) of genome-wide markers for MASLD in the discovery cohort among female participants. MASLD: metabolic dysfunction-associated steatotic liver disease; GWAS: genome-wide association study. [file 12967_2024_5478_MOESM2_ESM.pdf]

**A**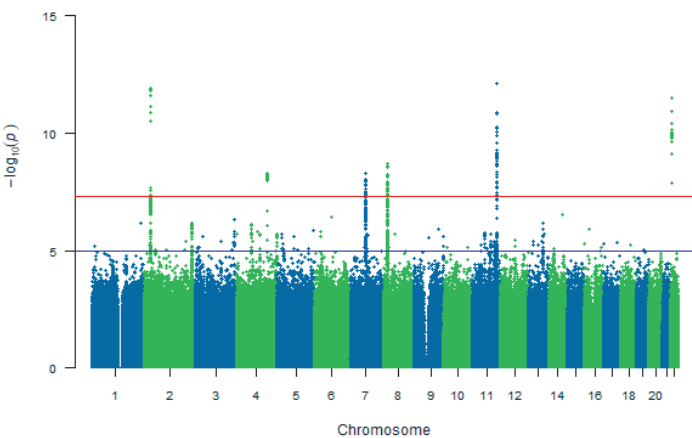**B**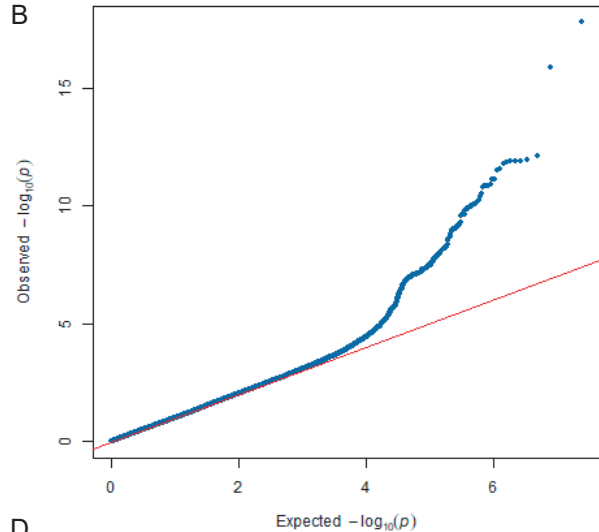**C**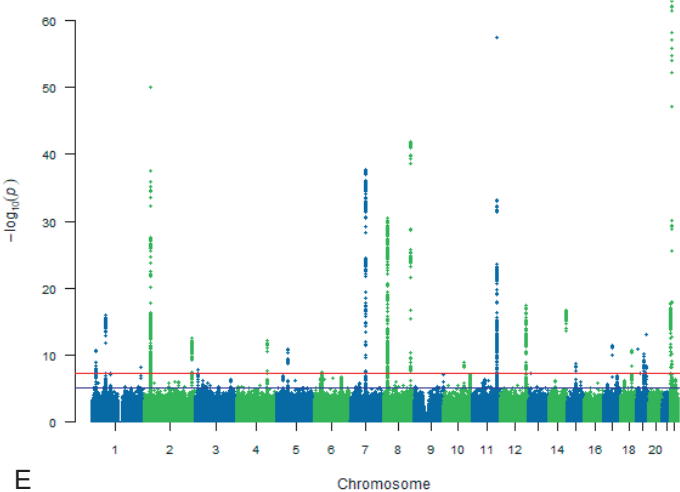**D**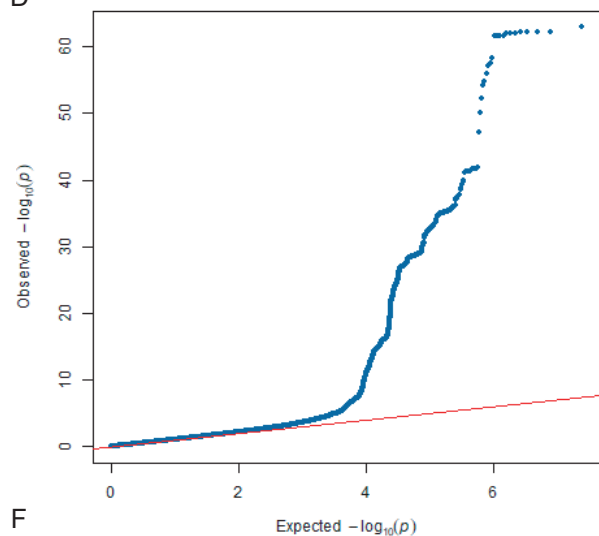**E**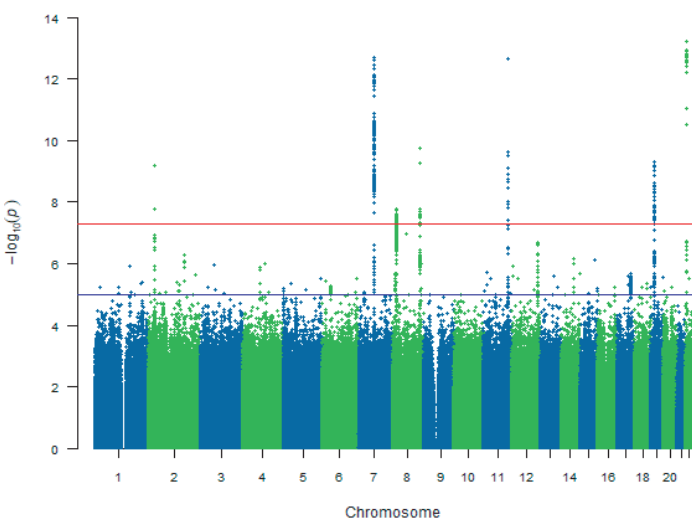**F**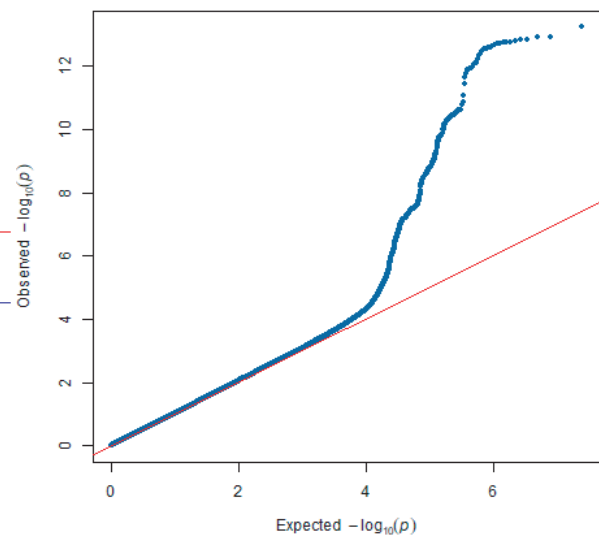

Supplement: Supplementary file 3 — Supplementary Material 3: Fig. S3. Results of GWAS case–control analysis within subgroups based on BMI categories. Manhattan plot (A) and Q–Q plot (B) of genome-wide markers for MASLD in the discovery cohort among the participants with normal BMI. Manhattan plot (C) and Q–Q plot (D) of genome-wide markers for MASLD in the discovery cohort among overweight participants. Manhattan plot (E) and Q–Q plot (F) of genome-wide markers for MASLD in the discovery cohort among obese participants. MASLD: metabolic dysfunction-associated steatotic liver disease; GWAS: genome-wide association study; Normal BMI: BMI < 25 kg/m2; overweight: 25 kg/m2 ≤ BMI < 30 kg/m2; obese: BMI ≥ 30 kg/m2. [file 12967_2024_5478_MOESM3_ESM.pdf]

A

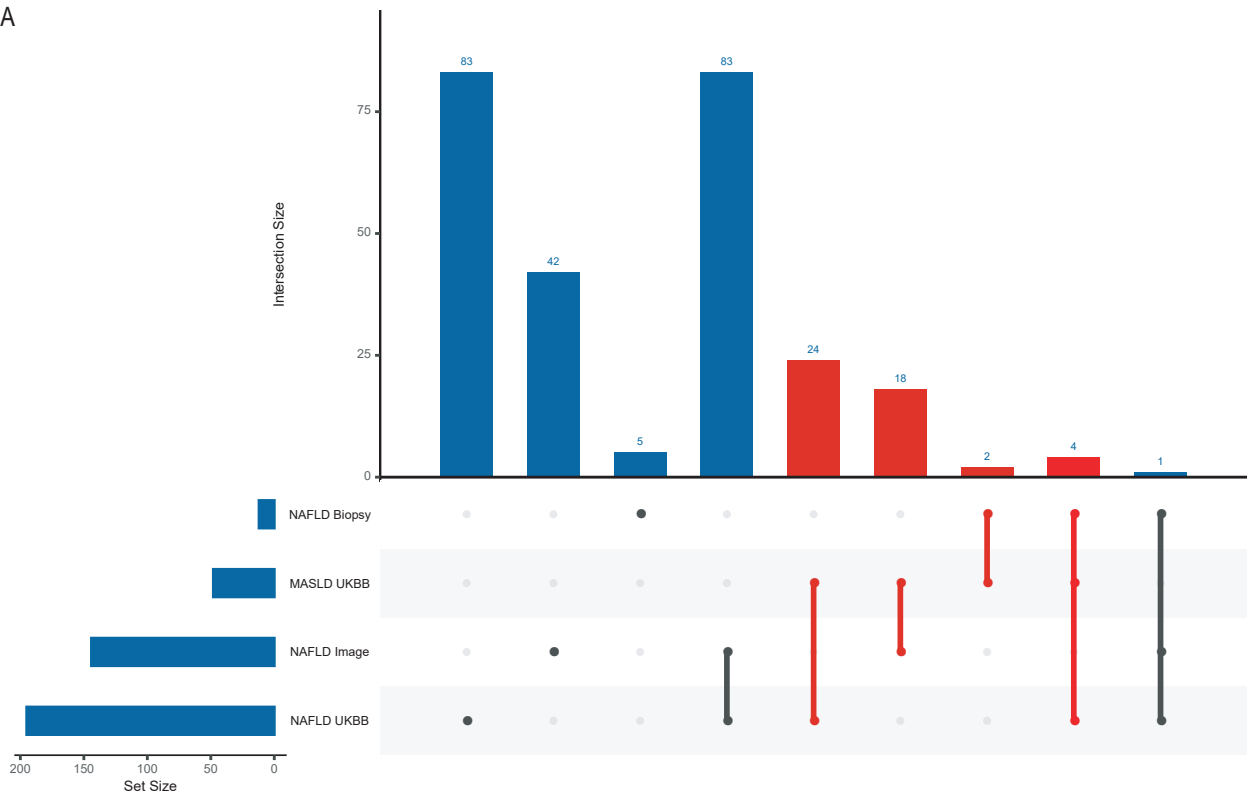

B

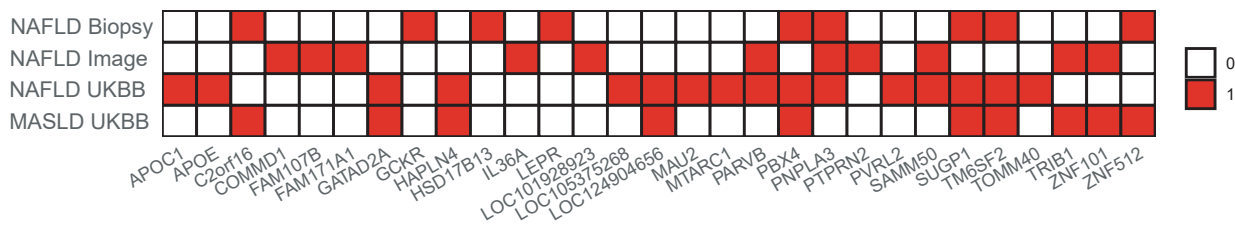

C

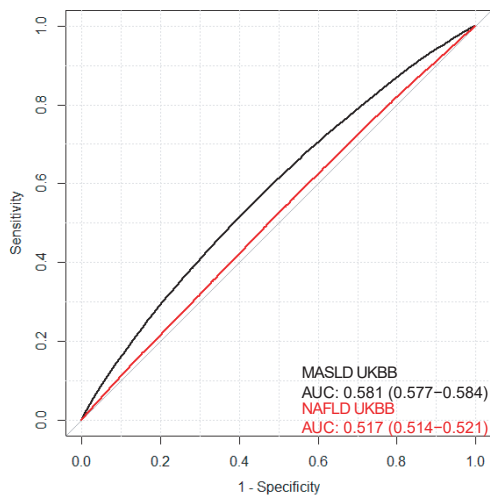

D

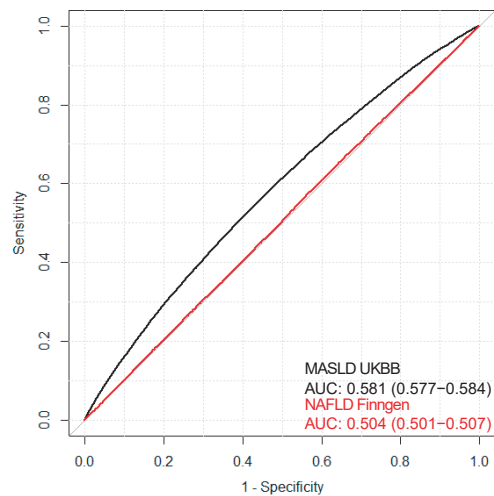

Supplement: Supplementary file 4 — Supplementary Material 4: Fig. S4. Comparison of GWAS and PRS results between MASLD and NAFLD. (A) The intersection of significant SNPs associated with MASLD and published SNPs associated with NAFLD. (B) Comparison of MASLD and NAFLD-related genes. (C) and (D) ROC graphical plot the diagnostic ability of different sources of PRS for MASLD in the replication cohort. NAFLD Biopsy: the summary statistics of GWAS for NAFLD diagnosed with biopsy; NAFLD Image: the summary statistics of GWAS for NAFLD diagnosed with image; NAFLD UKBB: Summary statistics of GWAS for NAFLD in the UKBB discovery cohort; MASLD UKBB: Summary statistics of the GWAS for MASLD in the discovery cohort of UKBB; MASLD: metabolic dysfunction-associated steatotic liver disease; GWAS: genome-wide association study; NAFLD: non-alcoholic fatty liver disease; UKBB: UK Biobank. [file 12967_2024_5478_MOESM4_ESM.pdf]

A

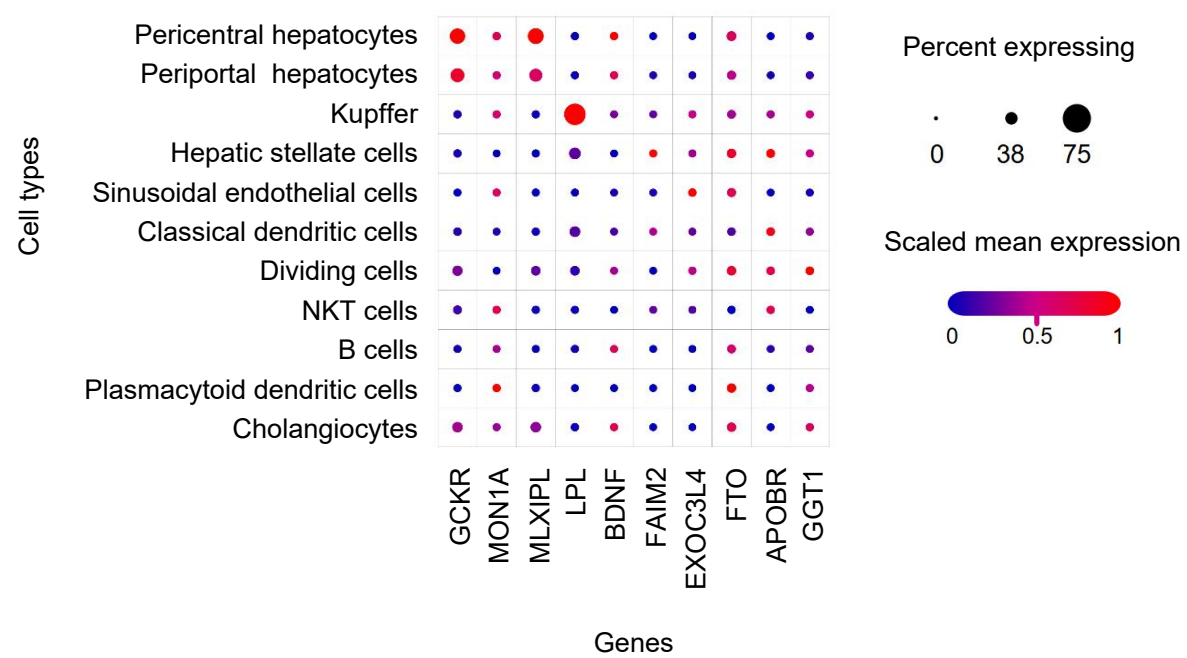

B

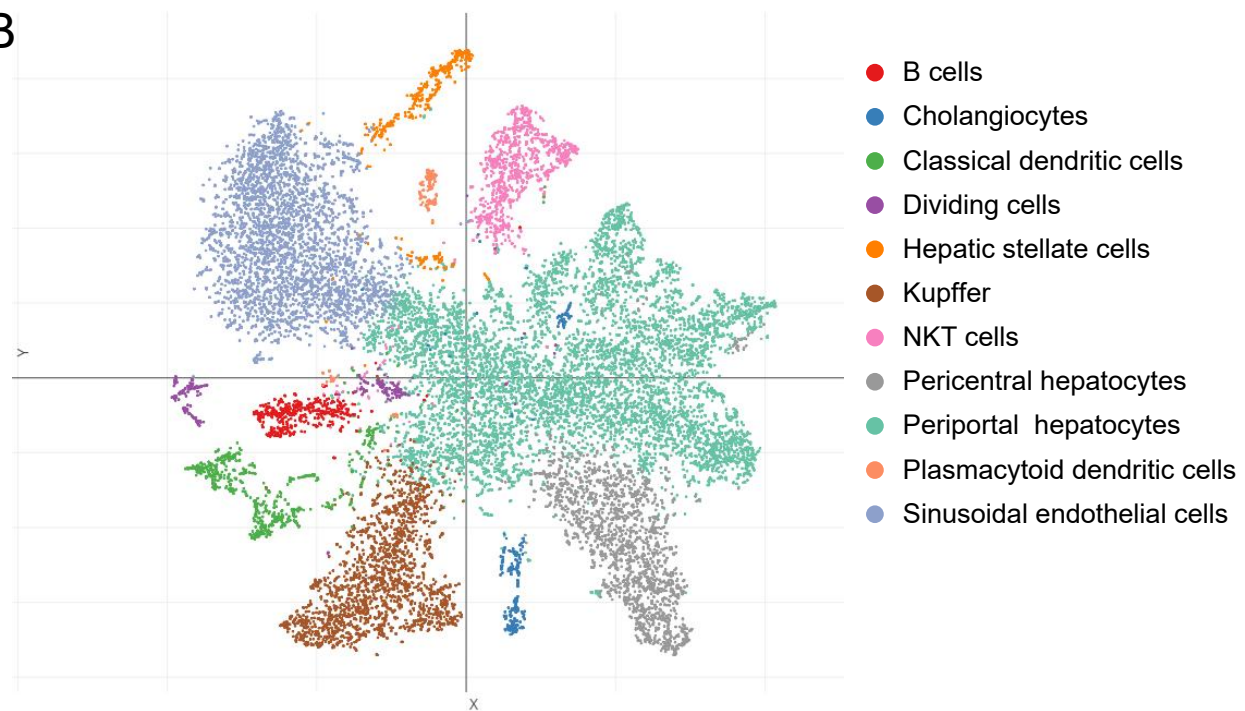

C

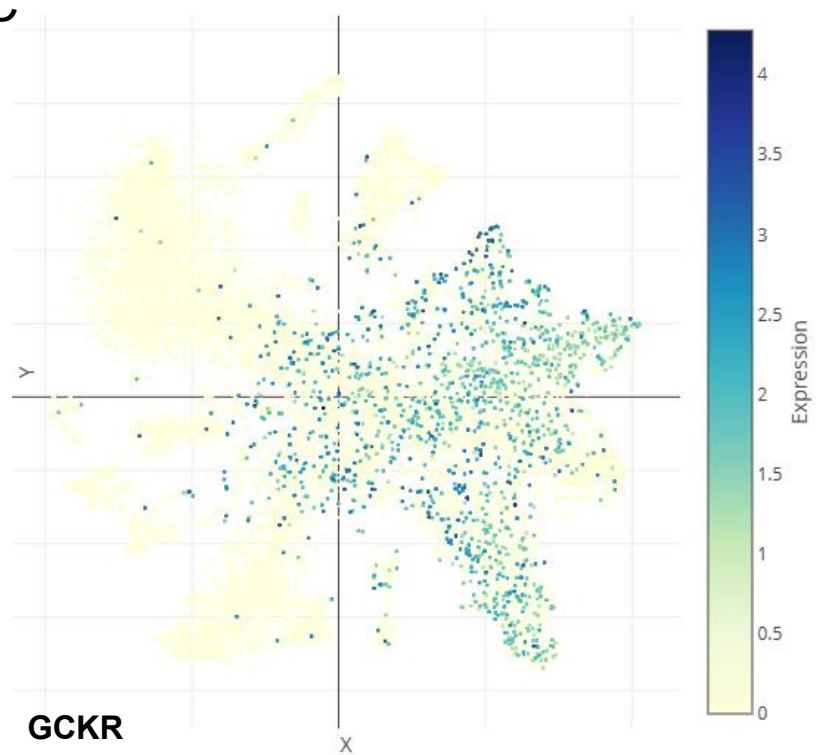

D

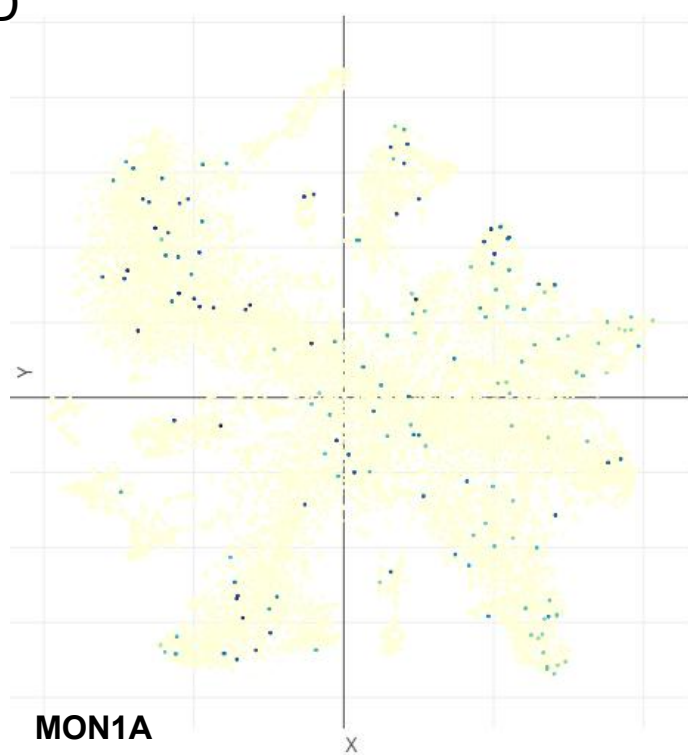

E

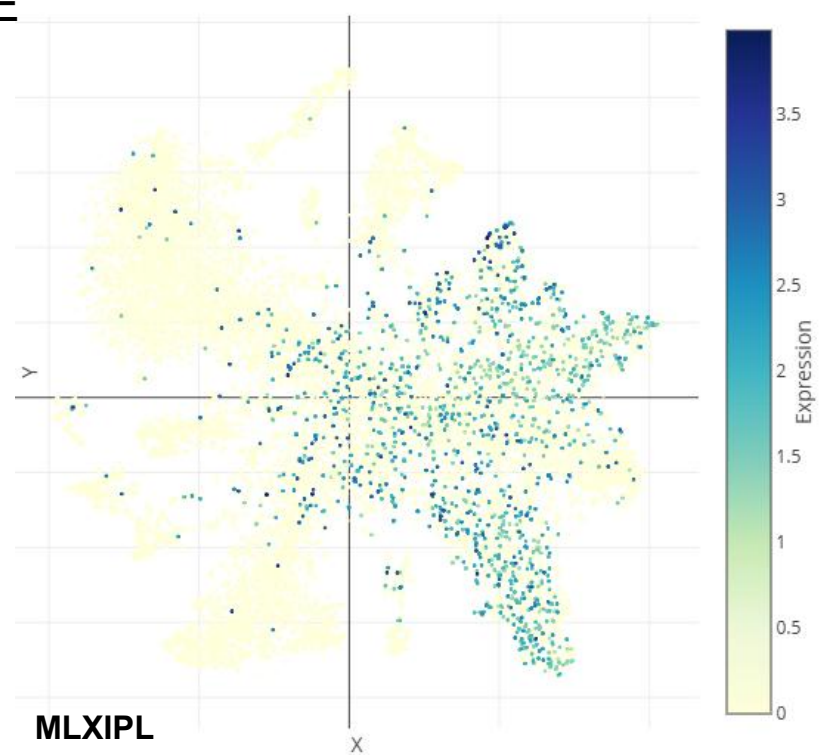

F

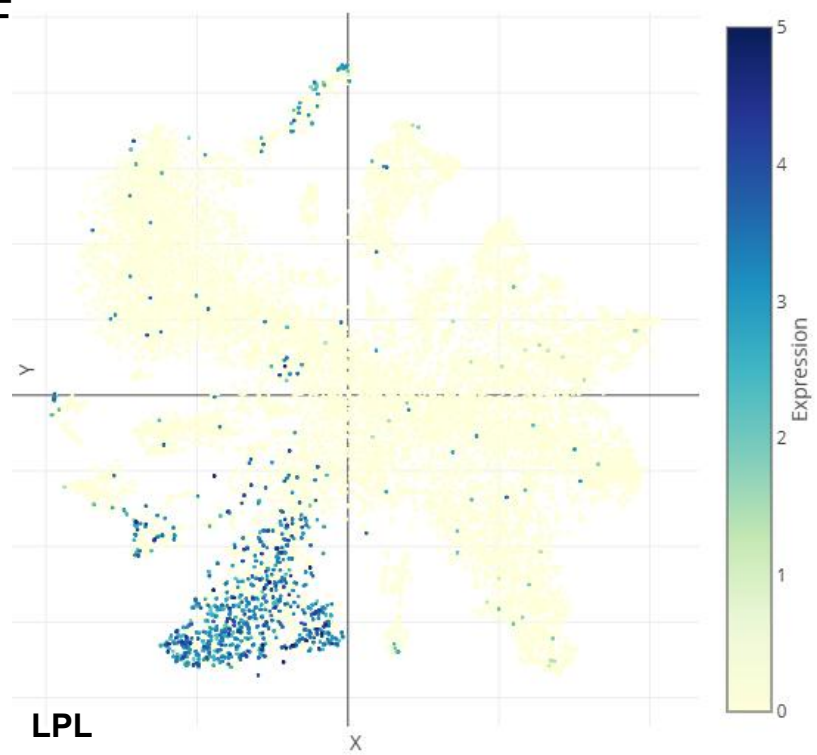

G

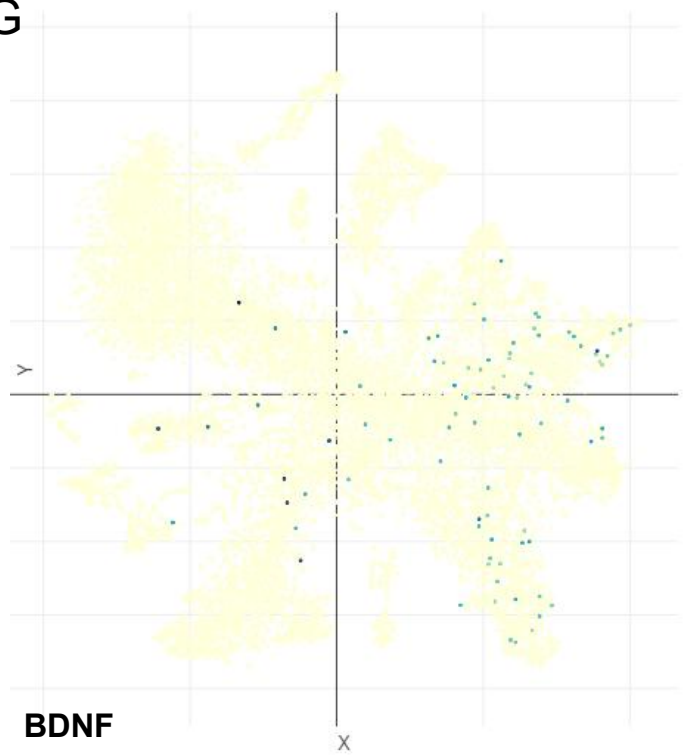

H

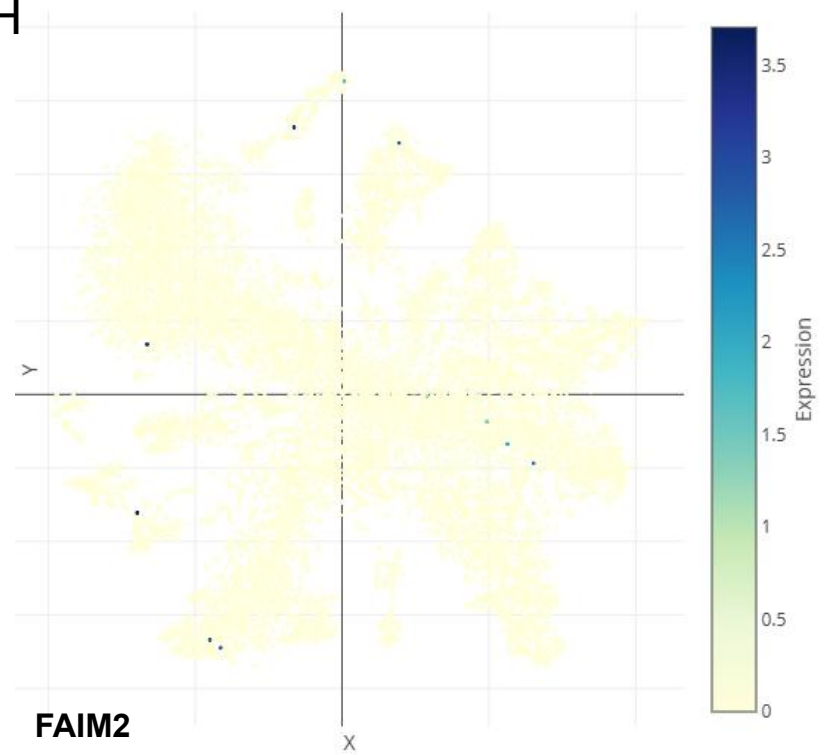

I

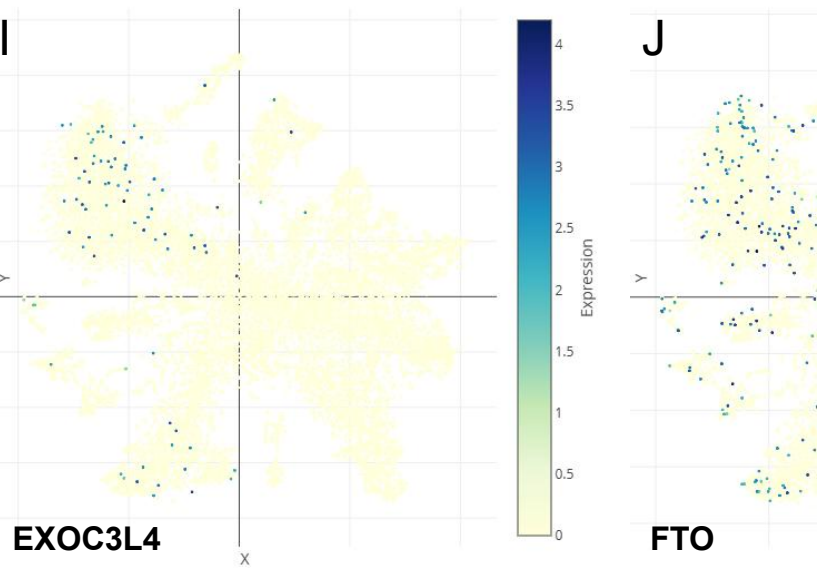

J

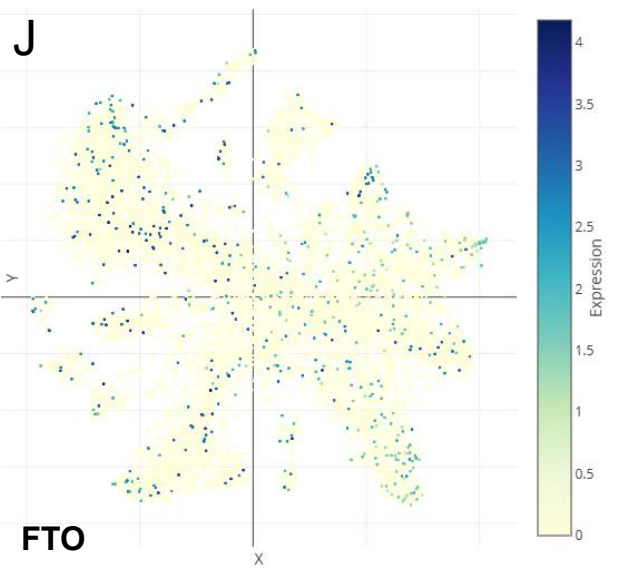

K

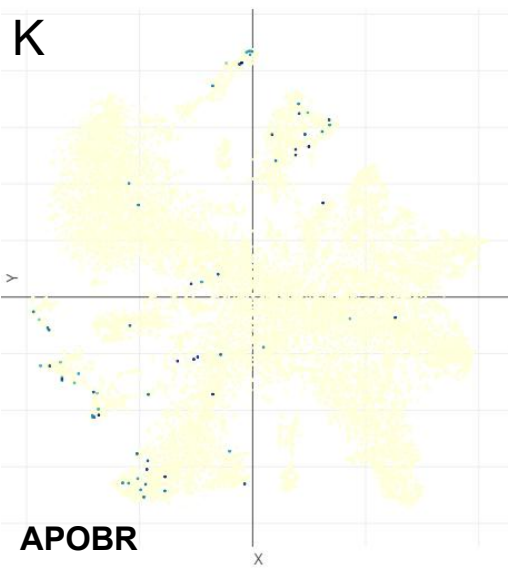

L

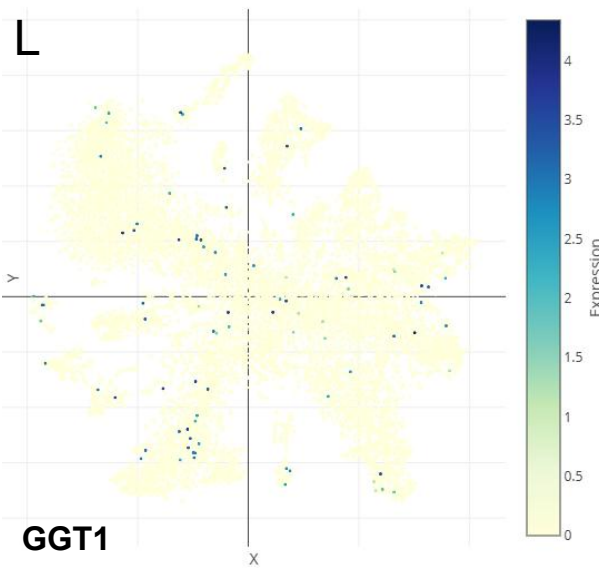

Supplement: Supplementary file 5 — Supplementary Material 5: Fig. S5. Cell type-specific expression of the genes identified in MASLD GWAS based on the single-cell RNA sequencing data from liver cells in metabolic syndrome mouse models. (A) Dot plots illustrating the expression of genes in each cell cluster. (B) UMAP plot of 11 cell clusters. (C–L) UMAP plots showing the expression of specific genes (GCKR, MON1A, MLXIPL, LPL, BDNF, FAIM2, EXOC3L4, FTO, APOBR, and GGT1) across the cell clusters. UMAP: Uniform Manifold Approximation and Projection. This analysis was conducted using online tools available at the Single Cell Portal. (https://singlecell.broadinstitute.org/single_cell/study/SCP1404/multitissue-single-cell-analysis-reveals-differential-tissue-cellular-and-molecular-sensitivity-between-fructose-and-high-fat-high-sucrose-diets-liver). [file 12967_2024_5478_MOESM5_ESM.pdf]
