# Supplementary material for: Polygenic risk score of metabolic dysfunction-associated steatotic liver disease amplifies the health impact on severe liver disease and metabolism-related outcomes
Source: J Transl Med. 2024 Jul 12;22:650. doi: 10.1186/s12967-024-05478-z (PMC11241780; doi:10.1186/s12967-024-05478-z)
Supplement: Supplementary file 6 — Supplementary Material 6: Table S1. Coding algorithms for defining diseases in the UK Biobank. [file 12967_2024_5478_MOESM6_ESM.docx]

Table S1. Coding Algorithms for Defining diseases in the UK Biobank

| Diseases | ICD-10 code | ICD-9 code | Others |
| --- | --- | --- | --- |
| Severe liver disease | C220, I850, I859, K703, K704, K721, K741, K742, K746, K766, K767, Z944 | 1550,4560,5308,4561,7476,4562A,5715,5712,571,570,5718,5713,5722,5719,5715,5723,5724, V427 |  |
| Type 2 Diabetes | E11 | 250, 2500, 2509, 6480, 7902 | Self-report: type 2 diabetes  Exclude: Type 1 Diabetes |
| Hypertension | I10, I11, I12, I13, I15, O10 | 401, 402, 403, 404, 405 | Medication: ACE inhibitors, AT-II antagonists, Beta-blockers, Calcium channel-blockers, Thiazide diuretics;  Self-report: hypertension, essential hypertension |
| Coronary artery disease | I21, I22, I23, I252, Z951 | 410, 412, 414 | Self-report: heart attack/myocardial infarction, coronary angioplasty +/- stent, cabg, triple heart bypass |
| Stroke | I607, I600, I609, I619, I611, I614, I613, I615, I616, I629, I630, I632, I635, I678, I64X, I690, I612, I693, I606, I608, I618, I638, I60, I61, I601, I602, I603, I604, I64, I633 | 430,431,7670,432,4329,  4340,4341, |  |
| Heart failure | I50, I110, I130, I132, Z941, T862 | 428 | Self-report: heart failure/pulmonary odema, heart transplant |
| Chronic kidney disease | I120, I131, I132, N18, N180, N181, N182, N183, N184, N185, N188, N189 | 585, 5859 |  |
| Non-alcoholic fatty liver disease | K740, K746, K758, K760 | 5715, 5718, 5719 | Exclude individuals with liver disease other than NAFLD |
