# Supplementary material for: Polygenic risk score of metabolic dysfunction-associated steatotic liver disease amplifies the health impact on severe liver disease and metabolism-related outcomes
Source: J Transl Med. 2024 Jul 12;22:650. doi: 10.1186/s12967-024-05478-z (PMC11241780; doi:10.1186/s12967-024-05478-z)
Supplement: Supplementary file 7 — Supplementary Material 7: Table S2. The associations of MASLD in different PRS group with SLD and extrahepatic outcomes. [file 12967_2024_5478_MOESM7_ESM.docx]

Table S2. The associations of MASLD in different PRS group with SLD and extrahepatic outcomes.

| Outcome | PRS Group | Events | Model1 | | Model2 | | Model3 | |
| --- | --- | --- | --- | --- | --- | --- | --- | --- |
|  |  |  | HR [95% CI] | *P* | HR [95% CI] | *P* | HR [95% CI] | *P* |
| SLD | Overall | 561 | 4.24 [3.62, 4.95] | <0.001 | 2.86 [2.34, 3.51] | <0.001 | 2.84 [2.31, 3.48] | <0.001 |
|  | Low | 212 | 3.73 [3.08, 4.50] | <0.001 | 2.55 [2.03, 3.19] | <0.001 | 2.58 [2.05, 3.23] | <0.001 |
|  | High | 349 | 4.62 [3.90, 5.47] | <0.001 | 3.15 [2.54, 3.90] | <0.001 | 3.07 [2.47, 3.82] | <0.001 |
| CAD | Overall | 4995 | 2.14 [2.06, 2.23] | <0.001 | 1.32 [1.25, 1.40] | <0.001 | 1.27 [1.20, 1.34] | <0.001 |
|  | Low | 2102 | 2.09 [1.98, 2.20] | <0.001 | 1.28 [1.20, 1.37] | <0.001 | 1.24 [1.16, 1.32] | <0.001 |
|  | High | 2893 | 2.18 [2.08, 2.29] | <0.001 | 1.36 [1.28, 1.44] | <0.001 | 1.30 [1.22, 1.38] | <0.001 |
| Stroke | Overall | 1383 | 2.28 [2.21, 2.36] | <0.001 | 1.23 [1.11, 1.37] | <0.001 | 1.18 [1.06, 1.30] | 0.002 |
|  | Low | 601 | 1.56 [1.42, 1.71] | <0.001 | 1.23 [1.09, 1.38] | 0.001 | 1.18 [1.05, 1.33] | 0.005 |
|  | High | 782 | 1.52 [1.40, 1.66] | <0.001 | 1.24 [1.11, 1.39] | <0.001 | 1.17 [1.04, 1.31] | 0.007 |
| HF | Overall | 2367 | 2.52 [2.36, 2.68] | <0.001 | 1.20 [1.10, 1.31] | <0.001 | 1.12 [1.02, 1.22] | 0.012 |
|  | Low | 971 | 2.40 [2.22, 2.60] | <0.001 | 1.16 [1.05, 1.27] | 0.003 | 1.09 [0.99, 1.20] | 0.076 |
|  | High | 1396 | 2.60 [2.42, 2.80] | <0.001 | 1.24 [1.13, 1.36] | <0.001 | 1.14 [1.04, 1.25] | 0.007 |
| Hypertension | Overall | 7497 | 2.28 [2.21, 2.36] | <0.001 | 1.41 [1.35, 1.47] | <0.001 | 1.36 [1.31, 1.43] | <0.001 |
|  | Low | 3208 | 2.24 [2.15, 2.33] | <0.001 | 1.40 [1.33, 1.47] | <0.001 | 1.38 [1.31, 1.45] | <0.001 |
|  | High | 4289 | 2.32 [2.23, 2.41] | <0.001 | 1.41 [1.35, 1.48] | <0.001 | 1.35 [1.29, 1.42] | <0.001 |
| CKD | Overall | 3042 | 2.37 [2.24, 2.50] | <0.001 | 1.33 [1.23, 1.43] | <0.001 | 1.20 [1.11, 1.30] | <0.001 |
|  | Low | 1212 | 2.19 [2.04, 2.35] | <0.001 | 1.24 [1.14, 1.35] | <0.001 | 1.15 [1.06, 1.25] | 0.001 |
|  | High | 1830 | 2.50 [2.35, 2.66] | <0.001 | 1.41 [1.30, 1.52] | <0.001 | 1.25 [1.15, 1.35] | <0.001 |
| T2DM | Overall | 5046 | 6.18 [5.83, 6.56] | <0.001 | 2.67 [2.49, 2.87] | <0.001 | 2.57 [2.39, 2.77] | <0.001 |
|  | Low | 2002 | 5.64 [5.27, 6.04] | <0.001 | 2.52 [2.33, 2.73] | <0.001 | 2.50 [2.31, 2.70] | <0.001 |
|  | High | 3044 | 6.60 [6.20, 7.03] | <0.001 | 2.81 [2.60, 3.03] | <0.001 | 2.64 [2.45, 2.85] | <0.001 |
| OS | Overall | 5024 | 1.69 [1.63, 1.76] | <0.001 | 1.23 [1.16, 1.30] | <0.001 | 1.20 [1.13, 1.26] | <0.001 |
|  | Low | 2116 | 1.66 [1.58, 1.75] | <0.001 | 1.19 [1.12, 1.27] | <0.001 | 1.17 [1.10, 1.25] | <0.001 |
|  | High | 2908 | 1.72 [1.64, 1.80] | <0.001 | 1.26 [1.19, 1.34] | <0.001 | 1.22 [1.14, 1.29] | <0.001 |

MASLD: metabolic dysfunction-associated steatotic liver disease; PRS: Polygenic Risk Score; SLD: severe liver disease; CAD, coronary artery disease; HF: Heart failure; CKD: Chronic kidney disease; T2DM: Type 2 diabetes; OS: overall survival; HR: Hazard ratio; CI: confidence interval;

Model 1 was unadjusted;

Model 2 was adjusted for sex, age at recruitment, genotyping chip and body mass index;

Model 3 was adjusted for Model 2 + hypoglycemic drugs + antihypertensive drugs + statins.
