# Supplementary material for: Polygenic risk score of metabolic dysfunction-associated steatotic liver disease amplifies the health impact on severe liver disease and metabolism-related outcomes
Source: J Transl Med. 2024 Jul 12;22:650. doi: 10.1186/s12967-024-05478-z (PMC11241780; doi:10.1186/s12967-024-05478-z)
Supplement: Supplementary file 9 — Supplementary Material 9: Table S4. The associations of top conditionally independent SNPs with overall survival in the whole cohort. [file 12967_2024_5478_MOESM9_ESM.docx]

| Table S4. The associations of top conditionally independent SNPs with overall survival in the whole cohort. | | | | | | |
| --- | --- | --- | --- | --- | --- | --- |
| SNP |  | Events | Model1 | | Model2 | |
|  |  |  | HR [95% CI] | *P* | HR [95% CI] | *P* |
|  | G/G | 200961 |  |  |  |  |
| rs11030108 | A/G | 188748 | 1.02 [1.00, 1.05] | 0.061 | 1.02 [1.00, 1.04] | 0.101 |
|  | A/A | 44795 | 1.02 [0.99, 1.06] | 0.221 | 1.02 [0.98, 1.05] | 0.412 |
|  | C/C | 24699 |  |  |  |  |
| rs964184 | G/C | 7455 | 0.98 [0.96, 1.01] | 0.155 | 0.99 [0.96, 1.01] | 0.258 |
|  | G/G | 585 | 1.00 [0.92, 1.08] | 0.936 | 0.99 [0.91, 1.07] | 0.797 |
|  | A/A | 4853 |  |  |  |  |
| rs7132908 | G/A | 15579 | 1.00 [0.97, 1.03] | 0.964 | 1.01 [0.98, 1.04] | 0.672 |
|  | G/G | 12307 | 0.99 [0.96, 1.02] | 0.532 | 1.00 [0.97, 1.03] | 0.925 |
|  | G/G | 6356 |  |  |  |  |
| rs2274685 | A/G | 15705 | 1.01 [0.98, 1.04] | 0.465 | 1.02 [0.99, 1.05] | 0.18 |
|  | A/A | 9385 | 0.99 [0.96, 1.03] | 0.688 | 1.00 [0.97, 1.03] | 0.944 |
|  | G/G | 6003 |  |  |  |  |
| rs40831 | A/G | 15802 | 0.99 [0.96, 1.02] | 0.376 | 0.99 [0.96, 1.02] | 0.456 |
|  | A/A | 10465 | 0.97 [0.94, 1.01] | 0.108 | 0.98 [0.95, 1.01] | 0.265 |
|  | A/A | 5899 |  |  |  |  |
| rs11075985 | C/A | 16106 | 1.00 [0.97, 1.03] | 0.777 | 1.03 [1.00, 1.06] | 0.087 |
|  | C/C | 10711 | 0.98 [0.95, 1.02] | 0.335 | 1.02 [0.98, 1.05] | 0.322 |
|  | G/G | 1771 |  |  |  |  |
| 18:57850927:GTCT:G | GTCT/G | 11723 | 1.01 [0.96, 1.06] | 0.66 | 1.02 [0.97, 1.07] | 0.438 |
|  | GTCT/GTCT | 19217 | 1.01 [0.96, 1.06] | 0.719 | 1.03 [0.98, 1.08] | 0.24 |
|  | GAT/GAT | 14 |  |  |  |  |
| rs538303513 | G/GAT | 1272 | 0.87 [0.51, 1.47] | 0.595 | 0.79 [0.47, 1.33] | 0.375 |
|  | G/G | 31339 | 0.88 [0.52, 1.48] | 0.619 | 0.78 [0.46, 1.32] | 0.361 |
|  | C/C | 65 |  |  |  |  |
| rs62106258 | T/C | 2861 | 1.11 [0.87, 1.42] | 0.391 | 1.09 [0.85, 1.39] | 0.512 |
|  | T/T | 29813 | 1.19 [0.93, 1.52] | 0.165 | 1.15 [0.90, 1.47] | 0.264 |
|  | C/C | 22567 |  |  |  |  |
| rs6731688 | A/C | 9132 | 0.98 [0.96, 1.01] | 0.135 | 0.99 [0.96, 1.01] | 0.278 |
|  | A/A | 951 | 0.98 [0.92, 1.05] | 0.619 | 1.00 [0.93, 1.06] | 0.916 |
|  | A/A | 12376 |  |  |  |  |
| 2:27748992:AT:A | AT/A | 14972 | 0.97 [0.95, 1.00] | 0.036 | 0.98 [0.96, 1.00] | 0.108 |
|  | AT/AT | 4622 | 0.96 [0.93, 1.00] | 0.034 | 0.97 [0.94, 1.00] | 0.095 |
|  | G/G | 3928 |  |  |  |  |
| rs3859862 | A/G | 14712 | 0.98 [0.95, 1.02] | 0.306 | 0.99 [0.95, 1.02] | 0.41 |
|  | A/A | 13990 | 0.98 [0.95, 1.02] | 0.3 | 0.98 [0.95, 1.02] | 0.346 |
|  | C/C | 22 |  |  |  |  |
| rs116946885 | A/C | 1544 | 0.94 [0.61, 1.43] | 0.761 | 0.96 [0.63, 1.46] | 0.841 |
|  | A/A | 30340 | 0.97 [0.64, 1.47] | 0.891 | 0.98 [0.65, 1.49] | 0.936 |
|  | C/C | 8880 |  |  |  |  |
| 3:49959570:CA:C | CA/C | 15841 | 0.99 [0.96, 1.01] | 0.257 | 0.99 [0.96, 1.01] | 0.353 |
|  | CA/CA | 6905 | 0.96 [0.93, 0.99] | 0.013 | 0.97 [0.94, 1.00] | 0.048 |
|  | T/T | 858 |  |  |  |  |
| rs17145750 | C/T | 9003 | 1.01 [0.94, 1.09] | 0.722 | 1.01 [0.94, 1.09] | 0.728 |
|  | C/C | 22878 | 0.98 [0.92, 1.05] | 0.658 | 0.99 [0.92, 1.06] | 0.687 |
|  | A/A | 2695 |  |  |  |  |
| rs2119690 | G/A | 13570 | 1.02 [0.98, 1.07] | 0.28 | 1.03 [0.99, 1.07] | 0.175 |
|  | G/G | 16435 | 1.00 [0.96, 1.04] | 0.881 | 1.01 [0.97, 1.05] | 0.568 |
| SNP: single-nucleotide polymorphism; HR: hazard ratio; CI: confidence interval Model 1 was unadjusted; Model 2 was adjusted for sex, age at recruitment, genotyping chip and body mass index; | | | | | | |
|  |  |  |  |  |  |  |
|  |  |  |  |  |  |  |
