# Supplementary material for: Polygenic risk score of metabolic dysfunction-associated steatotic liver disease amplifies the health impact on severe liver disease and metabolism-related outcomes
Source: J Transl Med. 2024 Jul 12;22:650. doi: 10.1186/s12967-024-05478-z (PMC11241780; doi:10.1186/s12967-024-05478-z)
Supplement: Supplementary file 10 — Supplementary Material 10: Table S5. The associations of top conditionally independent SNPs with overall survival in the MASLD group. [file 12967_2024_5478_MOESM10_ESM.docx]

| Table S5.The associations of top conditionally independent SNPs with overall survival in the MASLD group. | | | | | | |
| --- | --- | --- | --- | --- | --- | --- |
| SNP |  | Events | Model1 | | Model2 | |
|  |  |  | HR [95% CI] | *P* | HR [95% CI] | *P* |
|  | G/G | 7410 |  |  |  |  |
| rs11030108 | A/G | 7387 | 1.03 [1.00, 1.06] | 0.091 | 1.03 [1.00, 1.07] | 0.049 |
|  | A/A | 1797 | 1.03 [0.98, 1.08] | 0.292 | 1.03 [0.98, 1.09] | 0.227 |
|  | C/C | 12398 |  |  |  |  |
| rs964184 | G/C | 3890 | 0.95 [0.92, 0.99] | 0.011 | 0.97 [0.93, 1.00] | 0.084 |
|  | G/G | 332 | 0.99 [0.89, 1.10] | 0.847 | 1.00 [0.89, 1.11] | 0.947 |
|  | A/A | 2583 |  |  |  |  |
| rs7132908 | G/A | 7912 | 0.98 [0.94, 1.03] | 0.394 | 0.99 [0.94, 1.03] | 0.508 |
|  | G/G | 6125 | 0.97 [0.93, 1.01] | 0.174 | 0.97 [0.93, 1.02] | 0.264 |
|  | G/G | 3296 |  |  |  |  |
| rs2274685 | A/G | 8010 | 1.01 [0.97, 1.06] | 0.476 | 1.02 [0.98, 1.06] | 0.343 |
|  | A/A | 4646 | 1.00 [0.95, 1.04] | 0.915 | 1.00 [0.95, 1.04] | 0.848 |
|  | G/G | 3172 |  |  |  |  |
| rs40831 | A/G | 7992 | 0.97 [0.93, 1.01] | 0.106 | 0.97 [0.93, 1.01] | 0.111 |
|  | A/A | 5226 | 0.97 [0.93, 1.01] | 0.171 | 0.97 [0.93, 1.01] | 0.187 |
|  | A/A | 3209 |  |  |  |  |
| rs11075985 | C/A | 8155 | 1.00 [0.96, 1.04] | 0.946 | 1.01 [0.97, 1.06] | 0.495 |
|  | C/C | 5246 | 0.99 [0.94, 1.03] | 0.556 | 1.01 [0.96, 1.05] | 0.73 |
|  | G/G | 934 |  |  |  |  |
| 18:57850927:GTCT:G | GTCT/G | 6043 | 1.04 [0.97, 1.11] | 0.31 | 1.04 [0.97, 1.11] | 0.256 |
|  | GTCT/GTCT | 9629 | 1.05 [0.98, 1.12] | 0.175 | 1.06 [0.99, 1.13] | 0.11 |
|  | GAT/GAT | 4 |  |  |  |  |
| rs538303513 | G/GAT | 627 | 1.37 [0.51, 3.66] | 0.532 | 1.18 [0.44, 3.15] | 0.745 |
|  | G/G | 15932 | 1.30 [0.49, 3.46] | 0.603 | 1.10 [0.41, 2.94] | 0.842 |
|  | C/C | 29 |  |  |  |  |
| rs62106258 | T/C | 1346 | 1.05 [0.73, 1.52] | 0.787 | 1.07 [0.74, 1.54] | 0.728 |
|  | T/T | 15245 | 1.12 [0.78, 1.62] | 0.532 | 1.15 [0.80, 1.66] | 0.45 |
|  | C/C | 11548 |  |  |  |  |
| rs6731688 | A/C | 4560 | 1.00 [0.97, 1.04] | 0.848 | 0.99 [0.96, 1.03] | 0.701 |
|  | A/A | 459 | 1.00 [0.91, 1.10] | 0.947 | 1.01 [0.92, 1.11] | 0.838 |
|  | A/A | 6169 |  |  |  |  |
| 2:27748992:AT:A | AT/A | 7669 | 0.96 [0.93, 1.00] | 0.034 | 0.97 [0.94, 1.01] | 0.128 |
|  | AT/AT | 2409 | 0.94 [0.90, 0.98] | 0.009 | 0.96 [0.91, 1.00] | 0.055 |
|  | G/G | 2119 |  |  |  |  |
| rs3859862 | A/G | 7542 | 0.97 [0.93, 1.02] | 0.292 | 0.98 [0.94, 1.03] | 0.5 |
|  | A/A | 6904 | 0.99 [0.94, 1.04] | 0.654 | 0.99 [0.94, 1.03] | 0.56 |
|  | C/C | 4 |  |  |  |  |
| rs116946885 | A/C | 733 | 2.21 [0.83, 5.89] | 0.115 | 2.13 [0.80, 5.70] | 0.131 |
|  | A/A | 15462 | 2.22 [0.83, 5.93] | 0.11 | 2.16 [0.81, 5.75] | 0.124 |
|  | C/C | 4673 |  |  |  |  |
| 3:49959570:CA:C | CA/C | 7951 | 0.97 [0.93, 1.00] | 0.054 | 0.96 [0.93, 1.00] | 0.043 |
|  | CA/CA | 3413 | 0.96 [0.92, 1.00] | 0.062 | 0.95 [0.91, 0.99] | 0.029 |
|  | T/T | 403 |  |  |  |  |
| rs17145750 | C/T | 4483 | 1.02 [0.92, 1.13] | 0.704 | 1.04 [0.94, 1.15] | 0.494 |
|  | C/C | 11734 | 0.98 [0.89, 1.08] | 0.697 | 1.01 [0.91, 1.11] | 0.902 |
|  | A/A | 1322 |  |  |  |  |
| rs2119690 | G/A | 6741 | 1.01 [0.96, 1.08] | 0.625 | 1.03 [0.97, 1.10] | 0.288 |
|  | G/G | 8538 | 0.99 [0.94, 1.05] | 0.819 | 1.02 [0.96, 1.08] | 0.568 |
| SNP: single-nucleotide polymorphism; HR: hazard ratio; CI: confidence interval Model 1 was unadjusted; Model 2 was adjusted for sex, age at recruitment, genotyping chip and body mass index; | | | | | | |
|  |  |  |  |  |  |  |
|  |  |  |  |  |  |  |
