# Supplementary material for: Polygenic risk score of metabolic dysfunction-associated steatotic liver disease amplifies the health impact on severe liver disease and metabolism-related outcomes
Source: J Transl Med. 2024 Jul 12;22:650. doi: 10.1186/s12967-024-05478-z (PMC11241780; doi:10.1186/s12967-024-05478-z)
Supplement: Supplementary file 11 — Supplementary Material 11: Table S6. The associations of top conditionally independent SNPs with overall survival in the non-MASLD group. [file 12967_2024_5478_MOESM11_ESM.docx]

| Table S6. The associations of top conditionally independent SNPs with overall survival in the non-MASLD group. | | | | | | |
| --- | --- | --- | --- | --- | --- | --- |
| SNP |  | Events | Model1 | | Model2 | |
|  |  |  | HR [95% CI] | *P* | HR [95% CI] | *P* |
|  | G/G | 7531 |  |  |  |  |
| rs11030108 | A/G | 6948 | 1.00 [0.97, 1.04] | 0.89 | 1.01 [0.97, 1.04] | 0.715 |
|  | A/A | 1609 | 0.99 [0.94, 1.05] | 0.819 | 1.00 [0.95, 1.06] | 0.922 |
|  | C/C | 12301 |  |  |  |  |
| rs964184 | G/C | 3565 | 0.98 [0.95, 1.02] | 0.345 | 0.99 [0.95, 1.03] | 0.598 |
|  | G/G | 253 | 0.94 [0.83, 1.07] | 0.353 | 0.95 [0.84, 1.07] | 0.409 |
|  | A/A | 2270 |  |  |  |  |
| rs7132908 | G/A | 7667 | 1.03 [0.99, 1.08] | 0.175 | 1.03 [0.99, 1.08] | 0.184 |
|  | G/G | 6182 | 1.03 [0.98, 1.08] | 0.186 | 1.03 [0.98, 1.08] | 0.31 |
|  | G/G | 3060 |  |  |  |  |
| rs2274685 | A/G | 7695 | 1.02 [0.97, 1.06] | 0.463 | 1.02 [0.98, 1.07] | 0.292 |
|  | A/A | 4739 | 1.01 [0.97, 1.06] | 0.653 | 1.01 [0.97, 1.06] | 0.528 |
|  | G/G | 2831 |  |  |  |  |
| rs40831 | A/G | 7810 | 1.02 [0.98, 1.06] | 0.388 | 1.01 [0.97, 1.06] | 0.608 |
|  | A/A | 5239 | 1.00 [0.96, 1.05] | 0.928 | 0.99 [0.95, 1.04] | 0.825 |
|  | A/A | 2690 |  |  |  |  |
| rs11075985 | C/A | 7951 | 1.04 [1.00, 1.09] | 0.065 | 1.04 [1.00, 1.09] | 0.08 |
|  | C/C | 5465 | 1.03 [0.98, 1.08] | 0.205 | 1.03 [0.98, 1.07] | 0.286 |
|  | G/G | 837 |  |  |  |  |
| 18:57850927:GTCT:G | GTCT/G | 5680 | 1.01 [0.93, 1.08] | 0.889 | 1.00 [0.93, 1.07] | 0.991 |
|  | GTCT/GTCT | 9588 | 1.01 [0.94, 1.08] | 0.845 | 1.00 [0.93, 1.08] | 0.956 |
|  | GAT/GAT | 10 |  |  |  |  |
| rs538303513 | G/GAT | 645 | 0.64 [0.34, 1.20] | 0.166 | 0.64 [0.34, 1.19] | 0.157 |
|  | G/G | 15407 | 0.66 [0.36, 1.23] | 0.196 | 0.67 [0.36, 1.24] | 0.201 |
|  | C/C | 36 |  |  |  |  |
| rs62106258 | T/C | 1515 | 1.12 [0.81, 1.57] | 0.486 | 1.13 [0.81, 1.57] | 0.479 |
|  | T/T | 14568 | 1.16 [0.84, 1.61] | 0.37 | 1.17 [0.85, 1.63] | 0.335 |
|  | C/C | 11019 |  |  |  |  |
| rs6731688 | A/C | 4572 | 0.98 [0.95, 1.01] | 0.227 | 0.98 [0.94, 1.01] | 0.208 |
|  | A/A | 492 | 1.00 [0.91, 1.09] | 0.925 | 0.98 [0.89, 1.07] | 0.618 |
|  | A/A | 6207 |  |  |  |  |
| 2:27748992:AT:A | AT/A | 7303 | 0.97 [0.94, 1.00] | 0.076 | 0.98 [0.94, 1.01] | 0.181 |
|  | AT/AT | 2213 | 0.96 [0.92, 1.01] | 0.115 | 0.97 [0.93, 1.02] | 0.272 |
|  | G/G | 1809 |  |  |  |  |
| rs3859862 | A/G | 7170 | 1.01 [0.96, 1.06] | 0.703 | 1.00 [0.95, 1.05] | 0.956 |
|  | A/A | 7086 | 1.02 [0.97, 1.07] | 0.533 | 1.00 [0.95, 1.05] | 0.994 |
|  | C/C | 18 |  |  |  |  |
| rs116946885 | A/C | 811 | 0.63 [0.40, 1.01] | 0.055 | 0.68 [0.43, 1.09] | 0.109 |
|  | A/A | 14878 | 0.65 [0.41, 1.03] | 0.065 | 0.70 [0.44, 1.11] | 0.128 |
|  | C/C | 4207 |  |  |  |  |
| 3:49959570:CA:C | CA/C | 7890 | 1.02 [0.98, 1.06] | 0.338 | 1.01 [0.97, 1.05] | 0.536 |
|  | CA/CA | 3492 | 0.99 [0.95, 1.03] | 0.627 | 0.98 [0.94, 1.03] | 0.424 |
|  | T/T | 455 |  |  |  |  |
| rs17145750 | C/T | 4520 | 0.99 [0.90, 1.09] | 0.786 | 0.98 [0.89, 1.08] | 0.632 |
|  | C/C | 11144 | 0.95 [0.87, 1.05] | 0.325 | 0.94 [0.86, 1.04] | 0.227 |
|  | A/A | 1373 |  |  |  |  |
| rs2119690 | G/A | 6829 | 1.02 [0.97, 1.08] | 0.448 | 1.02 [0.97, 1.08] | 0.433 |
|  | G/G | 7897 | 0.98 [0.93, 1.04] | 0.604 | 1.00 [0.94, 1.06] | 0.934 |
| SNP: single-nucleotide polymorphism; HR: hazard ratio; CI: confidence interval Model 1 was unadjusted; Model 2 was adjusted for sex, age at recruitment, genotyping chip and body mass index; | | | | | | |
|  |  |  |  |  |  |  |
|  |  |  |  |  |  |  |
