# Supplementary material for: Polygenic risk score of metabolic dysfunction-associated steatotic liver disease amplifies the health impact on severe liver disease and metabolism-related outcomes
Source: J Transl Med. 2024 Jul 12;22:650. doi: 10.1186/s12967-024-05478-z (PMC11241780; doi:10.1186/s12967-024-05478-z)
Supplement: Supplementary file 12 — Supplementary Material 12: Table S7. Summary of conditionally independent SNPs in the MASLD case–control analysis among male participants. [file 12967_2024_5478_MOESM12_ESM.docx]

| **Table S7.** Summary of conditionally independent SNPs in the MASLD case-control analysis among male participants. | | | | | | | | |
| --- | --- | --- | --- | --- | --- | --- | --- | --- |
| SNP | Chr | POS | A1 | Nearest gene | Discovery cohort | | Replication cohort | |
|  |  |  |  |  | OR (95% CI) | P | OR (95% CI) | P |
| 1:16506926_CAA_C | 1 | 16506926 | C | - | 1.046 (1.03-1.063) | 7.577E-09 | 1.03 (1.006-1.054) | 1.509E-02 |
| rs10889356 | 1 | 63155349 | A | DOCK7 | 0.949 (0.934-0.964) | 9.525E-11 | 0.954 (0.931-0.977) | 1.191E-04 |
| rs2613503 | 1 | 72839774 | A | LOC105378797 | 1.057 (1.037-1.077) | 1.100E-08 | 1.037 (1.008-1.068) | 1.260E-02 |
| 2:27748992_AT_A | 2 | 27748992 | A | - | 0.941 (0.927-0.956) | 2.456E-14 | 0.951 (0.929-0.974) | 3.254E-05 |
| rs11387790 | 2 | 631396 | TA | - | 1.071 (1.05-1.092) | 1.314E-11 | 1.066 (1.034-1.099) | 3.544E-05 |
| rs62104180 | 2 | 466003 | A | - | 0.892 (0.862-0.923) | 7.716E-11 | 0.881 (0.836-0.928) | 2.090E-06 |
| rs6739755 | 2 | 59330227 | G | - | 0.949 (0.935-0.964) | 3.374E-11 | 0.975 (0.952-0.998) | 3.476E-02 |
| rs1046953 | 3 | 50197097 | T | SEMA3F | 0.955 (0.94-0.969) | 2.955E-09 | 0.963 (0.941-0.986) | 1.482E-03 |
| rs112820322 | 3 | 131625204 | G | CPNE4 | 0.958 (0.944-0.973) | 2.856E-08 | 0.975 (0.953-0.998) | 3.380E-02 |
| rs35321613 | 3 | 170665296 | C | - | 1.059 (1.042-1.076) | 4.725E-12 | 1.025 (1-1.05) | 5.481E-02 |
| rs78578284 | 4 | 3281868 | C | RGS12 | 1.069 (1.045-1.094) | 1.228E-08 | 1.015 (0.98-1.051) | 4.167E-01 |
| rs2161228 | 5 | 88001798 | T | MEF2C-AS2 | 1.076 (1.049-1.103) | 9.672E-09 | 1.064 (1.024-1.105) | 1.420E-03 |
| rs35100164 | 6 | 162963003 | C | PRKN | 0.936 (0.915-0.958) | 2.810E-08 | 0.98 (0.946-1.014) | 2.494E-01 |
| rs17145750 | 7 | 73026378 | T | MLXIPL | 0.911 (0.892-0.929) | 2.399E-19 | 0.928 (0.899-0.957) | 1.976E-06 |
| rs2980886 | 8 | 126480088 | A | TRIB1 | 0.936 (0.923-0.951) | 9.755E-18 | 0.931 (0.91-0.953) | 1.001E-09 |
| rs34669405 | 8 | 19929197 | T | LPL | 0.945 (0.929-0.961) | 3.880E-11 | 0.958 (0.933-0.982) | 9.252E-04 |
| rs35237252 | 8 | 19870271 | A | LPL | 0.923 (0.907-0.939) | 2.023E-19 | 0.917 (0.893-0.942) | 2.424E-10 |
| 10:126316133_GC_G | 10 | 126316133 | G | - | 1.048 (1.031-1.065) | 2.845E-08 | 1.029 (1.004-1.055) | 2.536E-02 |
| rs10767657 | 11 | 27668794 | C | BDNF-AS | 0.941 (0.926-0.956) | 1.312E-13 | 0.95 (0.926-0.973) | 4.006E-05 |
| rs559220724 | 11 | 116523568 | C | LINC02702 | 1.304 (1.197-1.421) | 1.417E-09 | 1.407 (1.232-1.608) | 5.217E-07 |
| rs964184 | 11 | 116648917 | C | ZPR1 | 0.861 (0.842-0.88) | 1.848E-40 | 0.869 (0.84-0.898) | 2.337E-16 |
| rs7132908 | 12 | 50263148 | A | FAIM2 | 1.044 (1.028-1.06) | 4.676E-08 | 1.052 (1.027-1.077) | 2.624E-05 |
| rs7970695 | 12 | 121423376 | A | HNF1A | 1.052 (1.036-1.069) | 1.046E-10 | 1.025 (1.001-1.05) | 4.199E-02 |
| rs2274685 | 14 | 103575070 | G | EXOC3L4 | 1.052 (1.036-1.068) | 1.210E-10 | 1.058 (1.034-1.083) | 2.101E-06 |
| rs11075985 | 16 | 53805207 | A | FTO | 1.089 (1.073-1.106) | 1.699E-28 | 1.082 (1.057-1.107) | 2.799E-11 |
| rs12934406 | 16 | 29982566 | T | TMEM219 | 1.05 (1.035-1.066) | 1.798E-10 | 1.048 (1.024-1.072) | 6.963E-05 |
| rs4783721 | 16 | 69561156 | A | NFAT5 | 0.949 (0.934-0.963) | 1.474E-11 | 0.963 (0.941-0.986) | 1.473E-03 |
| rs62058015 | 16 | 59312392 | G | - | 1.044 (1.028-1.061) | 4.427E-08 | 0.992 (0.969-1.016) | 4.987E-01 |
| rs55931203 | 17 | 65854602 | T | BPTF | 1.068 (1.047-1.089) | 6.432E-11 | 1.045 (1.014-1.077) | 3.974E-03 |
| rs72836561 | 17 | 41926126 | T | CD300LG | 1.139 (1.091-1.19) | 3.285E-09 | 1.043 (0.977-1.114) | 2.029E-01 |
| rs10871755 | 18 | 56094783 | G | MIR122 | 0.949 (0.932-0.967) | 2.676E-08 | 0.955 (0.929-0.983) | 1.402E-03 |
| rs1808579 | 18 | 21104888 | T | NPC1 | 0.95 (0.936-0.965) | 3.635E-11 | 0.96 (0.938-0.982) | 4.818E-04 |
| rs182719967 | 18 | 58099814 | A | MC4R | 0.87 (0.83-0.912) | 6.053E-09 | 0.968 (0.9-1.041) | 3.828E-01 |
| rs66723169 | 18 | 57808978 | A | - | 1.064 (1.045-1.083) | 1.418E-11 | 1.067 (1.038-1.097) | 3.548E-06 |
| rs8097306 | 18 | 45640653 | A | ZBTB7C | 1.045 (1.029-1.061) | 2.301E-08 | 1.013 (0.99-1.037) | 2.670E-01 |
| rs190712692 | 19 | 45425178 | A | APOE | 1.118 (1.079-1.158) | 5.418E-10 | 1.093 (1.035-1.154) | 1.306E-03 |
| rs3859862 | 22 | 24997070 | G | GGT1 | 1.113 (1.096-1.131) | 2.763E-40 | 1.103 (1.077-1.13) | 1.303E-15 |
| rs5992136 | 22 | 18453103 | C | MICAL3 | 0.944 (0.928-0.96) | 3.468E-11 | 0.96 (0.935-0.985) | 1.874E-03 |

SNP: single-nucleotide polymorphism; Chr: chromosome; POS: position; OR: odds ratio; CI: confidence interval
