# Supplementary material for: Polygenic risk score of metabolic dysfunction-associated steatotic liver disease amplifies the health impact on severe liver disease and metabolism-related outcomes
Source: J Transl Med. 2024 Jul 12;22:650. doi: 10.1186/s12967-024-05478-z (PMC11241780; doi:10.1186/s12967-024-05478-z)
Supplement: Supplementary file 13 — Supplementary Material 13: Table S8. Summary of conditionally independent SNPs in the MASLD case–control analysis among female participants. [file 12967_2024_5478_MOESM13_ESM.docx]

| Table S8. Summary of conditionally independent SNPs in the MASLD case-control analysis among female participants. | | | | | | | | |
| --- | --- | --- | --- | --- | --- | --- | --- | --- |
| SNP | Chr | POS | A1 | Nearest gene | Discovery cohort | | Replication cohort | |
|  |  |  |  |  | OR (95% CI) | *P* | OR (95% CI) | *P* |
| rs147206915 | 1 | 155105388 | TTTTG | EFNA1 | 0.942 (0.927-0.957) | 5.055E-13 | 0.954 (0.931-0.978) | 1.973E-04 |
| rs4970720 | 1 | 98326447 | A | DPYD | 0.946 (0.928-0.964) | 1.831E-08 | 0.953 (0.925-0.981) | 1.350E-03 |
| rs545608 | 1 | 177899121 | C | SEC16B | 1.097 (1.076-1.119) | 3.514E-20 | 1.084 (1.052-1.117) | 1.390E-07 |
| rs768234885 | 1 | 62927741 | C | DOCK7 | 0.952 (0.936-0.968) | 1.334E-08 | 0.972 (0.947-0.997) | 2.943E-02 |
| 2:27748992_AT_A | 2 | 27748992 | A | - | 0.934 (0.918-0.949) | 4.571E-16 | 0.938 (0.915-0.962) | 6.683E-07 |
| rs10182036 | 2 | 25071835 | A | ADCY3 | 1.091 (1.06-1.123) | 4.099E-09 | 1.038 (0.994-1.085) | 9.069E-02 |
| rs188978973 | 2 | 70286082 | T | ASPRV1 | 1.409 (1.248-1.592) | 3.383E-08 | 0.916 (0.765-1.096) | 3.377E-01 |
| rs4671328 | 2 | 58935282 | G | LINC01122 | 0.951 (0.935-0.966) | 1.040E-09 | 0.971 (0.947-0.995) | 1.869E-02 |
| rs62106258 | 2 | 417167 | C | LOC124905962 | 0.858 (0.827-0.89) | 4.682E-16 | 0.833 (0.787-0.881) | 1.788E-10 |
| rs6731688 | 2 | 630662 | C | - | 1.089 (1.067-1.113) | 2.330E-15 | 1.075 (1.041-1.11) | 9.796E-06 |
| rs74262333 | 2 | 99393272 | C | MGAT4A | 1.097 (1.061-1.133) | 3.720E-08 | 0.989 (0.941-1.039) | 6.631E-01 |
| rs35418151 | 3 | 136162621 | C | STAG1 | 1.065 (1.046-1.085) | 7.835E-12 | 1.05 (1.022-1.079) | 4.939E-04 |
| rs57800857 | 4 | 140863365 | C | MAML3 | 0.954 (0.938-0.97) | 3.422E-08 | 0.969 (0.945-0.994) | 1.454E-02 |
| rs2443038 | 5 | 40964557 | C | C7 | 0.926 (0.901-0.95) | 1.101E-08 | 1.041 (1-1.083) | 5.006E-02 |
| rs6864091 | 5 | 75010002 | C | POC5 | 0.942 (0.926-0.958) | 1.459E-12 | 0.948 (0.925-0.973) | 3.836E-05 |
| rs4711961 | 6 | 50833084 | A | TFAP2B | 1.064 (1.042-1.087) | 8.965E-09 | 1.052 (1.019-1.086) | 2.090E-03 |
| rs1547958 | 7 | 150640285 | T | TMEM176A | 0.948 (0.93-0.965) | 1.216E-08 | 0.978 (0.951-1.006) | 1.157E-01 |
| rs2119690 | 8 | 19859539 | A | LPL | 0.936 (0.92-0.953) | 1.941E-13 | 0.96 (0.935-0.986) | 2.820E-03 |
| rs1064939 | 11 | 118396331 | T | KMT2A/TTC36/TTC36 | 0.831 (0.786-0.878) | 4.198E-11 | 0.931 (0.856-1.012) | 9.143E-02 |
| rs768994653 | 11 | 43877204 | C | - | 1.053 (1.035-1.072) | 1.086E-08 | 1.033 (1.006-1.061) | 1.824E-02 |
| rs964184 | 11 | 116648917 | C | ZPR1 | 0.917 (0.895-0.938) | 2.846E-13 | 0.902 (0.87-0.934) | 1.052E-08 |
| rs118091512 | 12 | 52239411 | T | FIGNL2 | 1.118 (1.077-1.161) | 5.661E-09 | 1.026 (0.969-1.086) | 3.810E-01 |
| rs9569813 | 13 | 58660568 | G | PCDH17 | 0.949 (0.931-0.967) | 4.831E-08 | 0.981 (0.953-1.009) | 1.858E-01 |
| 15:60887884_GA_G | 15 | 60887884 | G | - | 1.059 (1.042-1.077) | 1.215E-11 | 1.027 (1.002-1.054) | 3.452E-02 |
| rs10694251 | 16 | 69720803 | GTAAA | NFAT5 | 1.048 (1.031-1.065) | 2.610E-08 | 1.044 (1.018-1.07) | 6.627E-04 |
| rs11075985 | 16 | 53805207 | A | FTO | 1.104 (1.087-1.122) | 1.786E-33 | 1.1 (1.073-1.127) | 2.255E-14 |
| rs563738354 | 16 | 72271340 | TA | PMFBP1 | 1.175 (1.113-1.24) | 4.623E-09 | 0.966 (0.89-1.049) | 4.130E-01 |
| rs35146367 | 17 | 65831328 | TA | BPTF | 1.062 (1.041-1.084) | 4.867E-09 | 1.076 (1.044-1.11) | 2.934E-06 |
| 18:57850927_GTCT_G | 18 | 57850927 | G | - | 1.095 (1.075-1.116) | 3.453E-21 | 1.105 (1.074-1.137) | 4.740E-12 |
| rs11907932 | 20 | 51148656 | G | LOC105372666 | 0.946 (0.93-0.963) | 2.744E-10 | 0.993 (0.967-1.019) | 5.916E-01 |
| rs548684773 | 21 | 46437772 | TA | - | 0.899 (0.87-0.928) | 1.000E-10 | 0.996 (0.949-1.047) | 8.874E-01 |
| rs9612664 | 22 | 24995044 | A | GGT1 | 1.086 (1.068-1.105) | 5.996E-22 | 1.086 (1.059-1.114) | 2.167E-10 |

SNP: single-nucleotide polymorphism; Chr: chromosome; POS: position; OR: odds ratio; CI: confidence interval
