# Supplementary material for: Polygenic risk score of metabolic dysfunction-associated steatotic liver disease amplifies the health impact on severe liver disease and metabolism-related outcomes
Source: J Transl Med. 2024 Jul 12;22:650. doi: 10.1186/s12967-024-05478-z (PMC11241780; doi:10.1186/s12967-024-05478-z)
Supplement: Supplementary file 14 — Supplementary Material 14: Table S9. Summary of conditionally independent SNPs in the MASLD case–control analysis among participants with normal BMI. [file 12967_2024_5478_MOESM14_ESM.docx]

| Table S9. Summary of conditionally independent SNPs in the MASLD case-control analysis among participants with normal BMI | | | | | | | | |
| --- | --- | --- | --- | --- | --- | --- | --- | --- |
| SNP | Chr | POS | A1 | Nearest gene | Discovery cohort | | Replication cohort | |
|  |  |  |  |  | OR (95% CI) | *P* | OR (95% CI) | *P* |
| 2:27748992_AT_A | 2 | 27748992 | A | - | 0.786 (0.742-0.832) | 1.237E-16 | 0.861 (0.788-0.94) | 8.825E-04 |
| rs7669911 | 4 | 146789485 | T | ZNF827 | 1.249 (1.159-1.346) | 5.286E-09 | 1.198 (1.068-1.343) | 2.066E-03 |
| rs17145750 | 7 | 73026378 | T | MLXIPL | 0.798 (0.74-0.861) | 4.925E-09 | 0.799 (0.711-0.897) | 1.478E-04 |
| rs13275622 | 8 | 19943219 | A | LOC105379311 | 0.843 (0.797-0.891) | 2.049E-09 | 0.97 (0.89-1.057) | 4.837E-01 |
| rs964184 | 11 | 116648917 | C | ZPR1 | 0.69 (0.635-0.749) | 1.595E-18 | 0.646 (0.568-0.736) | 3.542E-11 |
| rs5751904 | 22 | 25000229 | T | GGT1 | 1.227 (1.158-1.3) | 3.088E-12 | 1.318 (1.206-1.441) | 1.245E-09 |
| SNP: single-nucleotide polymorphism; Chr: chromosome; POS: position; OR: odds ratio; CI: confidence interval | | | | | | | | |
