# Supplementary material for: Polygenic risk score of metabolic dysfunction-associated steatotic liver disease amplifies the health impact on severe liver disease and metabolism-related outcomes
Source: J Transl Med. 2024 Jul 12;22:650. doi: 10.1186/s12967-024-05478-z (PMC11241780; doi:10.1186/s12967-024-05478-z)
Supplement: Supplementary file 15 — Supplementary Material 15: Table S10. Summary of conditionally independent SNPs in the MASLD case–control analysis among overweight participants. [file 12967_2024_5478_MOESM15_ESM.docx]

| Table S10. Summary of conditionally independent SNPs in the MASLD case-control analysis among overweight participants. | | | | | | | | |
| --- | --- | --- | --- | --- | --- | --- | --- | --- |
| SNP | Chr | POS | A1 | Nearest gene | Discovery cohort | | Replication cohort | |
|  |  |  |  |  | OR (95% CI) | *P* | OR (95% CI) | *P* |
| 1:16506926_CAA_C | 1 | 16506926 | C | - | 1.058 (1.041-1.076) | 2.088E-11 | 1.044 (1.018-1.07) | 8.737E-04 |
| rs4846944 | 1 | 230395886 | G | GALNT2 | 0.928 (0.904-0.952) | 6.845E-09 | 0.97 (0.933-1.008) | 1.182E-01 |
| rs7547965 | 1 | 62926371 | G | DOCK7 | 0.932 (0.916-0.947) | 1.262E-16 | 0.949 (0.924-0.973) | 5.789E-05 |
| 2:27748992_AT_A | 2 | 27748992 | A | - | 0.881 (0.866-0.895) | 8.468E-51 | 0.888 (0.866-0.911) | 9.098E-20 |
| rs2396316 | 2 | 227133527 | T | LOC646736 | 1.065 (1.047-1.084) | 2.478E-13 | 1.044 (1.018-1.072) | 9.992E-04 |
| rs527620413 | 3 | 12360357 | GT | - | 0.931 (0.909-0.955) | 1.809E-08 | 0.963 (0.927-1) | 4.844E-02 |
| rs4547811 | 4 | 146794621 | C | ZNF827 | 1.082 (1.059-1.106) | 6.472E-13 | 1.097 (1.062-1.134) | 3.581E-08 |
| 5:55860907_GC_G | 5 | 55860907 | G | - | 1.074 (1.052-1.096) | 1.261E-11 | 1.041 (1.009-1.074) | 1.265E-02 |
| rs115177000 | 6 | 34203893 | A | HMGA1/LOC124901225 | 1.115 (1.073-1.159) | 2.978E-08 | 1.084 (1.022-1.151) | 7.370E-03 |
| rs2395227 | 6 | 32605935 | T | HLA-DQA1 | 0.938 (0.917-0.959) | 3.057E-08 | 0.92 (0.888-0.952) | 2.012E-06 |
| rs13234378 | 7 | 73026151 | T | MLXIPL | 0.853 (0.833-0.874) | 1.804E-38 | 0.854 (0.824-0.886) | 1.632E-17 |
| rs10106652 | 8 | 19928160 | A | LPL | 0.927 (0.911-0.944) | 1.916E-16 | 0.935 (0.909-0.961) | 1.663E-06 |
| rs17321515 | 8 | 126486409 | G | TRIB1 | 0.894 (0.88-0.909) | 1.495E-42 | 0.895 (0.873-0.917) | 5.084E-19 |
| rs2119690 | 8 | 19859539 | A | LPL | 0.9 (0.885-0.916) | 3.615E-31 | 0.922 (0.897-0.947) | 3.544E-09 |
| rs10882140 | 10 | 94842213 | T | CYP26A1 | 0.952 (0.936-0.967) | 1.496E-09 | 0.978 (0.954-1.002) | 7.605E-02 |
| rs140587667 | 11 | 116891007 | G | SIK3 | 1.421 (1.297-1.558) | 6.462E-14 | 1.429 (1.246-1.64) | 3.327E-07 |
| rs964184 | 11 | 116648917 | C | ZPR1 | 0.824 (0.804-0.843) | 3.309E-58 | 0.839 (0.81-0.87) | 1.146E-21 |
| rs1169289 | 12 | 121416622 | G | HNF1A | 0.93 (0.915-0.945) | 3.846E-18 | 0.962 (0.938-0.986) | 2.198E-03 |
| rs863750 | 12 | 124505444 | T | ZNF664-RFLNA | 1.049 (1.032-1.067) | 9.987E-09 | 1.037 (1.011-1.063) | 5.207E-03 |
| rs61462345 | 14 | 103564935 | G | EXOC3L4 | 1.084 (1.064-1.104) | 2.426E-17 | 1.06 (1.03-1.09) | 5.935E-05 |
| 15:60887884_GA_G | 15 | 60887884 | G | - | 1.053 (1.035-1.07) | 1.962E-09 | 1.044 (1.018-1.071) | 8.669E-04 |
| rs77697917 | 17 | 41840849 | T | EXOC3L4 | 1.185 (1.13-1.244) | 4.471E-12 | 1.109 (1.031-1.193) | 5.451E-03 |
| rs9959832 | 18 | 56086820 | T | MIR122,MIR3591 | 0.934 (0.915-0.953) | 1.921E-11 | 0.922 (0.894-0.95) | 1.888E-07 |
| 19:39281071_TTTTG_T | 19 | 39281071 | T | - | 1.055 (1.035-1.076) | 4.077E-08 | 1.006 (0.977-1.036) | 6.866E-01 |
| rs116843064 | 19 | 8429323 | A | ANGPTL4 | 0.816 (0.77-0.866) | 1.302E-11 | 0.776 (0.711-0.848) | 1.980E-08 |
| rs3786920 | 19 | 33994417 | C | PEPD | 0.946 (0.931-0.962) | 7.324E-11 | 0.972 (0.948-0.997) | 2.675E-02 |
| rs484195 | 19 | 45421877 | G | APOC1 | 1.067 (1.049-1.085) | 7.018E-14 | 1.03 (1.004-1.058) | 2.288E-02 |
| rs144548059 | 22 | 18480841 | GTGAA | MICAL3 | 0.918 (0.9-0.935) | 1.517E-18 | 0.947 (0.919-0.975) | 2.380E-04 |
| rs148246158 | 22 | 25037371 | T | BCRP3 | 0.794 (0.755-0.836) | 9.089E-19 | 0.828 (0.765-0.896) | 2.644E-06 |
| rs186765281 | 22 | 25010855 | G | GGT1 | 0.785 (0.736-0.837) | 1.693E-13 | 0.882 (0.8-0.974) | 1.274E-02 |
| rs3859862 | 22 | 24997070 | G | GGT1 | 1.156 (1.137-1.176) | 1.285E-63 | 1.156 (1.127-1.186) | 2.875E-28 |
| SNP: single-nucleotide polymorphism; Chr: chromosome; POS: position; OR: odds ratio; CI: confidence interval | | | | | | | | |
