# Supplementary material for: Polygenic risk score of metabolic dysfunction-associated steatotic liver disease amplifies the health impact on severe liver disease and metabolism-related outcomes
Source: J Transl Med. 2024 Jul 12;22:650. doi: 10.1186/s12967-024-05478-z (PMC11241780; doi:10.1186/s12967-024-05478-z)
Supplement: Supplementary file 16 — Supplementary Material 16: Table S11. Summary of conditionally independent SNPs in the MASLD case–control analysis among obese participants. [file 12967_2024_5478_MOESM16_ESM.docx]

| Table S11. Summary of conditionally independent SNPs in the MASLD case-control analysis among obese participants. | | | | | | | | |
| --- | --- | --- | --- | --- | --- | --- | --- | --- |
| SNP | Chr | POS | A1 | Nearest gene | Discovery cohort | | Replication cohort | |
|  |  |  |  |  | OR (95% CI) | *P* | OR (95% CI) | *P* |
| 2:27748992_AT_A | 2 | 27748992 | A |  | 0.894 (0.863-0.927) | 6.370E-10 | 0.847 (0.802-0.895) | 2.808E-09 |
| rs79624003 | 7 | 73012785 | G | MLXIPL | 0.827 (0.786-0.87) | 1.948E-13 | 0.838 (0.775-0.906) | 8.182E-06 |
| rs15285 | 8 | 19824667 | T | LPL | 0.898 (0.865-0.932) | 1.625E-08 | 0.918 (0.866-0.974) | 4.367E-03 |
| rs2954021 | 8 | 126482077 | G | TRIB1 | 0.896 (0.866-0.927) | 1.854E-10 | 0.923 (0.876-0.973) | 2.751E-03 |
| rs964184 | 11 | 116648917 | C | ZPR1 | 0.828 (0.787-0.871) | 2.197E-13 | 0.873 (0.809-0.942) | 4.963E-04 |
| rs3794991 | 19 | 19610596 | T | GATAD2A | 0.821 (0.771-0.873) | 4.989E-10 | 0.832 (0.756-0.916) | 1.706E-04 |
| rs3859862 | 22 | 24997070 | G | GGT1 | 1.147 (1.107-1.189) | 5.924E-14 | 1.143 (1.081-1.208) | 2.360E-06 |
| SNP: single-nucleotide polymorphism; Chr: chromosome; POS: position; OR: odds ratio; CI: confidence interval | | | | | | | | |
