# Supplementary material for: Polygenic risk score of metabolic dysfunction-associated steatotic liver disease amplifies the health impact on severe liver disease and metabolism-related outcomes
Source: J Transl Med. 2024 Jul 12;22:650. doi: 10.1186/s12967-024-05478-z (PMC11241780; doi:10.1186/s12967-024-05478-z)
Supplement: Supplementary file 17 — Supplementary Material 17: Table S12. Characteristics of NAFLD GWAS included in our study. [file 12967_2024_5478_MOESM17_ESM.docx]

Table S12. Characteristics of NAFLD GWAS Included in our study

|  | NAFLD Biopsy | NAFLD Image | NAFLD UKBB |
| --- | --- | --- | --- |
| PMID | 32298765 | 31311600 | Not published |
| Population | European | European | European |
| First author, Year | Quentin M Anstee, 202 | Bahram Namjou, 2019 | Not published |
| Sample size | 17,781 controls and 1483 cases | 8571 controls and 1106 cases | 453911 controls and 6073 cases |
| NAFLD assessment | Liver biopsy | Imaging or histology | ICD-10 and ICD-9 |

GWAS, genome-wide association study; ICD-9, International Classification of Diseases–Ninth Revision; ICD-10, International Classification of Diseases–Tenth Revision; NAFLD, nonalcoholic fatty liver disease;
